# A latitudinal phylogeographic diversity gradient in birds

**DOI:** 10.1371/journal.pbio.2001073

**Published:** 2017-04-13

**Authors:** Brian Tilston Smith, Glenn F. Seeholzer, Michael G. Harvey, Andrés M. Cuervo, Robb T. Brumfield

**Affiliations:** 1 Department of Ornithology, American Museum of Natural History, New York, New York, United States of America; 2 Museum of Natural Science and Department of Biological Sciences, Louisiana State University, Baton Rouge, Louisiana, United States of America; 3 Department of Ecology and Evolutionary Biology and Museum of Zoology, University of Michigan, Ann Arbor, Michigan, United States of America; 4 Department of Ecology and Evolutionary Biology, Tulane University, New Orleans, Louisiana, United States of America; Australian National University, Australia

## Abstract

High tropical species diversity is often attributed to evolutionary dynamics over long timescales. It is possible, however, that latitudinal variation in diversification begins when divergence occurs within species. Phylogeographic data capture this initial stage of diversification in which populations become geographically isolated and begin to differentiate genetically. There is limited understanding of the broader implications of intraspecific diversification because comparative analyses have focused on species inhabiting and evolving in restricted regions and environments. Here, we scale comparative phylogeography up to the hemisphere level and examine whether the processes driving latitudinal differences in species diversity are also evident within species. We collected genetic data for 210 New World bird species distributed across a broad latitudinal gradient and estimated a suite of metrics characterizing phylogeographic history. We found that lower latitude species had, on average, greater phylogeographic diversity than higher latitude species and that intraspecific diversity showed evidence of greater persistence in the tropics. Factors associated with species ecologies, life histories, and habitats explained little of the variation in phylogeographic structure across the latitudinal gradient. Our results suggest that the latitudinal gradient in species richness originates, at least partly, from population-level processes within species and are consistent with hypotheses implicating age and environmental stability in the formation of diversity gradients. Comparative phylogeographic analyses scaled up to large geographic regions and hundreds of species can show connections between population-level processes and broad-scale species-richness patterns.

## Author summary

The causes of high tropical species diversity remain contentious and disputed. Recent studies have shown that latitudinal differences in speciation and extinction rates give rise to high tropical diversity. However, it is unclear if this gradient is the product of population-level, species-level, or clade-level processes. Here, we used genetic, environmental, and morphological data from hundreds of bird species occurring in the Western Hemisphere to evaluate patterns of within-species diversity. We found that tropical species have greater intraspecific genetic variation, and this diversity persists longer than in temperate species. These results indicate that biodiversity gradients can arise more rapidly than previously thought and that the processes governing these gradients operate on multiple evolutionary timescales.

## Introduction

Phylogeographic studies leverage spatial and genetic data to examine the earliest stages of speciation, illuminating how populations differentiate across a landscape [1]. Comparisons across species show that the level of genetic structuring varies from deep phylogeographic breaks to unstructured panmictic populations [2]. This among-species variation in the amount and depth of phylogeographic structuring has been attributed to various factors, including differences in dispersal ability [3,4], habitat preferences [5], breeding phenology [6], life history traits [7], and the amount of evolutionary time in the landscape [8,9]. Because comparative phylogeographic studies usually examine species that occur within the same geographic region and in similar environmental and historical settings, the generality of associations between species traits and phylogeographic variation is largely unknown. Scaling phylogeography beyond the analysis of codistributed species and expanding comparative tests to multiple geographic assemblages of species would provide insight into whether the origins of genetic diversity link to large-scale biodiversity patterns.

The latitudinal gradient in species richness is one of the most ubiquitous ecological patterns in nature [10]. Phylogenetic data suggest that higher tropical species richness is attributable to a multitude of factors, including higher long-term diversification rates [11,12], niche conservatism [13], and more time for speciation [e.g., 14]. Latitudinal variation in phylogeographic structure is poorly understood, even though differences in diversification patterns among temperate and tropical clades could begin accumulating within species. Genetic divergence among populations has been shown to be higher in the tropics [15], but divergence patterns in subspecies [16] and sister species [17,18] suggest that there is faster diversification in the temperate zone. Processes that result in greater population differentiation and/or less extinction over phylogeographic timescales would result in greater intraspecific diversity within a region, the effects of which could persist to deeper phylogenetic scales. Assuming intraspecific genetic diversity varies among regions [e.g., 19], comparative analysis of phylogeographic structure between the temperate and tropical zones should provide insight into the formation of a more general latitudinal biodiversity gradient.

Habitat, landscape heterogeneity, and species dispersal ability are expected to influence phylogeographic structuring by determining how fragmented species distributions are and levels of gene flow between populations. These interactions among populations, however, may function differently across habitat types and regions. In tropical forests, higher available energy [20] and increased niche specialization along elevational gradients [21] and vertical habitat strata [22] are expected to lead to higher speciation rates. Greater climatic instability in temperate habitats during Pleistocene glacial—interglacial cycles has been linked to dynamic species histories of population expansion and contraction, higher extinction rates [23], and in some cases higher speciation rates [e.g., 17]. Species ecologies that differ between habitats and regions can also lead to differences in phylogeographic diversity. For example, genetic subdivision is often deeper in tropical species with lower dispersal abilities [4,7]. It is unclear if these traits have the same phylogeographic effect on temperate species, for which movements may be determined by changing climatic conditions more than intrinsic dispersal ability. Another possibility is that phylogeographic structuring may be random with respect to both the environment and ecology, such that the accumulation of genetic variation within a species may be due to its evolutionary persistence in the landscape [8]. The effects of environment, ecology, and persistence on species phylogeography are not mutually exclusive and may mediate broad patterns of intraspecific diversity among regions.

Here, we used hemisphere-scale intraspecific data from New World birds to test whether phylogeographic diversity varies from the temperate region to the tropics. We compiled a large multispecies dataset of mitochondrial DNA (mtDNA) with environmental, trait, and morphological data for each species. The bird species included in this study inhabit various ecoregions, including tropical lowland and montane rainforests, deserts, temperate deciduous forests, and montane pine forests. We used a Bayesian coalescent model to quantify the degree of phylogeographic structure from each species’ time-calibrated mtDNA gene tree. To test for latitudinal biodiversity gradients below the species level, we asked whether or not the degree of phylogeographic structure declined with increasing latitude. We additionally assessed and accounted for the relative influence of a range of variables characterizing environmental preferences, contemporary and historical environmental conditions, life history, morphology, and range sizes (S1 Table) on phylogeographic diversity. Finally, we evaluated possible population-level mechanisms for an intraspecific diversity gradient by assessing whether metrics of the rates of formation and loss of phylogeographic structure in species are tied to latitude. By characterizing broad-scale patterns of phylogeographic diversity and investigating their environmental, ecological, historical, and population-level causes, we provide powerful insight into how biodiversity patterns form at the early stages of diversification.

## Results

### An intraspecific latitudinal diversity gradient exists

**Fig 1:**
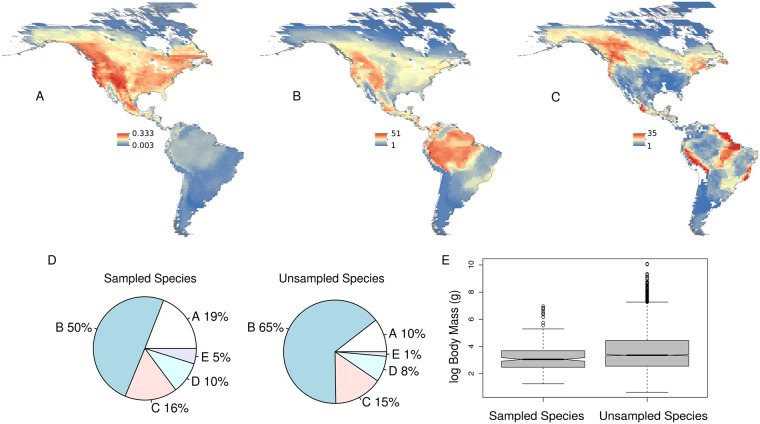
Summary of ecological and geographical sampling of the species used in this study. Shown is the proportion of species sampled with phylogeographic data out of the total diversity of terrestrial New World birds (A), species richness of those species that were sampled in this study (B), and unsampled areas within each species’ distribution (C). Pie charts (D) show foraging guilds of species sampled in this study versus unsampled species. Foraging guilds are as follows—A: Frugivore-Nectarivore, B: Insectivore, C: Omnivore, D: Granivore-Herbivore, E: Carnivore-Piscivore. The box plots (E) show log body masses (g) of the species sampled and unsampled in this study. The unsampled species represent all terrestrial New World birds. Shown are the first, second, and third quartiles; the lines are the 95% confidence intervals, and the circles represent outliers. Underlying data for 1DE is from the EltonTraits database [25].

We assembled mtDNA data from 17,573 individuals, representing 210 species, for which we collected environmental data from 67,779 observational records and morphological data from 1,139 museum specimens. The proportion of species sampled compared to the total diversity was higher in temperate North America versus tropical South America (Fig 1A), but the number of species sampled was higher in the tropics (Fig 1B). Species occurring in the highest latitude areas, such as temperate South America, were underrepresented in our dataset. Genetic sampling within species was poorest in areas shown in red (Fig 1C); for example, the northern limits of species occurring in Canada are undersampled. Our sampling of foraging guilds (Fig 1D) and body sizes (Fig 1E) qualitatively reflected their relative diversities with respect to unsampled species, except that very large birds are underrepresented in phylogeographic studies. To estimate phylogeographic structure, we required a metric that was comparable across species with varying degrees of sampling. The multispecies coalescent provides a framework for measuring genetic structure within species, and we used a Bayesian implementation demonstrated to perform well with variable sampling [24]. We found, on average, that our focal species (*n* = 210; S2 Table) included multiple genetic clusters (mean = 2.710; standard deviation [SD] = 2.310; range: 1:17) that were geographically structured.

**Fig 2:**
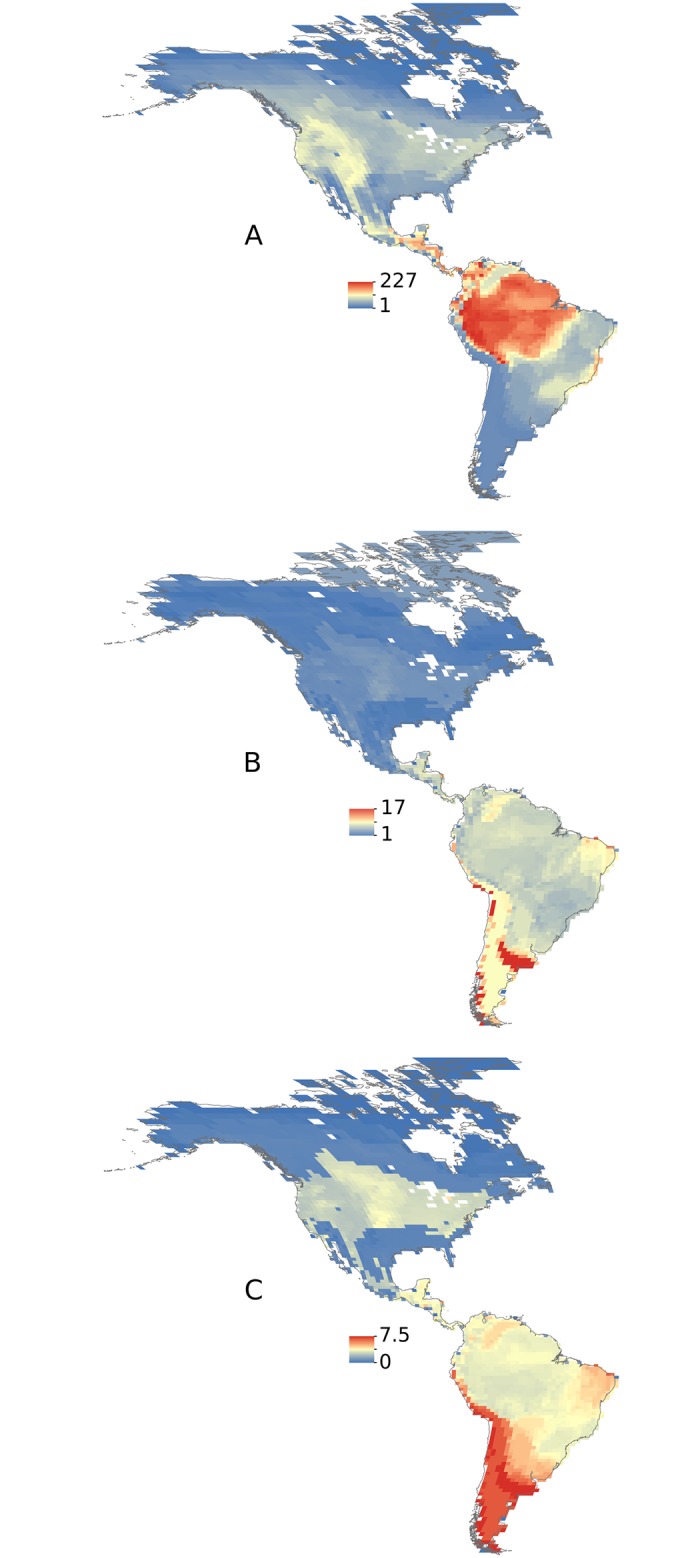
Species occurring in the tropics have more phylogeographic structure than those in the temperate zone. Heat maps show the per-pixel value of the summed (A), mean (B), and standard deviation (C) for phylogeographic structure across all species. Phylogeographic structure estimates are based on genetic clusters, identified using coalescent modeling (0.9 posterior probability threshold). Warmer colors denote higher values.

Using phylogenetic generalized least-squares (PGLS) analysis, we evaluated the association between phylogeographic structure and latitude while accounting for phylogenetic nonindependence. We used multivariate models to account for alternative explanatory variables characterizing species ecologies, life histories, and habitats. The explanatory powers of the multivariate models were compared using a version of the Akaike information criterion (AICc). The latitudinal midpoint of species’ distributions was a significant effect in the model, after accounting for all other variables (S3 Table). Because the model also included the age of each species, the latitudinal trend in phylogeographic structure is not merely attributable to latitudinal variation in species ages (S3 Table). Decreasing phylogeographic structure with latitude was more pronounced when we included species age based on stem age (ΔAICc = 16.059), the timing of divergence from the last common ancestor, as a covariate versus crown age (ΔAICc = 3.163), the time when all haplotypes coalesce within a species. The ΔAICc shows the change in the AICc score between the full model and a model excluding the predictor, with a ΔAICc > 2 considered a significant factor in the model. The models also accounted for sampling bias linked to the proportion of each species range sampled, which had a stronger effect in the model using stem age (ΔAICc = 2.862) versus crown age (ΔAICc = 1.353). Heat maps that reflect the phylogeographic structure across species occurring in each pixel provide a visual evaluation of trends across regions (Fig 2A–2C). The mean (Fig 2B) and SD (Fig 2C) of phylogeographic structure are higher in species occurring at lower latitudes in the tropics.

### The gradient is robust to several types of bias

**Fig 3:**
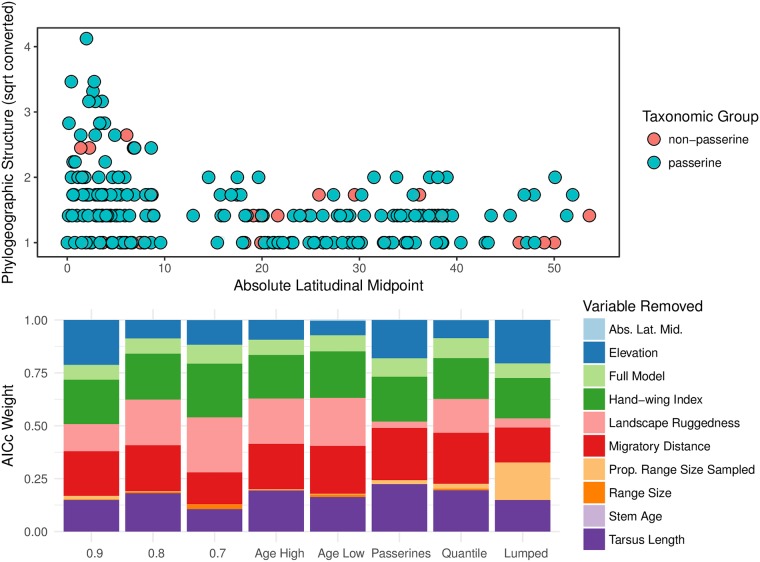
The phylogeographic diversity gradient in New Word birds is robust to several types of bias. In the top graph, the *y*-axis shows the amount of phylogeographic structure (square root converted), and the *x*-axis shows the absolute latitudinal midpoint in each species. Non-passerine and passerine species are denoted in rose and aqua, respectively. Phylogeographic structure increases towards the equator (top graph). The stability of the association between phylogeographic structure and latitude is shown with Akaike information criterion (AICc) weights, which were qualitatively similar across treatments assessing sources of potential bias (bottom graph). On the bottom graph, the *y*-axis shows the AICc weight for a particular multivariate-phylogenetic generalized least-squares (PGLS) model in which a variable was removed or all variables were included (Full Model). The thicker the bar for a particular variable, the less influence removal of that variable had on model fit. The *x*-axis shows the different bias treatments. Starting from the left, the column labeled “0.9” is the standard treatment using currently recognized species by the American Ornithological Society (AOS), and “0.8” and “0.7” are more liberal thresholds for inferring the degree of phylogeographic structure in each species. “Age High and Low” used the high and low of the 95% highest posterior density for species ages, respectively. We excluded non-passerines and species that were outliers with respect to phylogeographic structure (greater than the 95% quantile) in the “Passerines” and “Quantile” datasets, respectively. The “Lumped” dataset is an alternative taxonomy in which we combined allopatric or parapatric populations and species that formed a monophyletic group. Our PGLS analyses were robust to using alternative datasets to account for these different sources of bias. Underlying data can be found in S2 Table (top) and S3 Table (bottom).

We assessed whether higher phylogeographic structure in tropical species was an artifact of different sources of bias (Fig 3). Our PGLS analyses were robust to using an alternative taxonomy, examining passerines only (*n* = 178), excluding species that were outliers with respect to phylogeographic structure (*n* = 199; excluding structure estimates greater than the 95% quantile), and uncertainty in estimates of population structure or species age (Fig 3 bottom; S3 Table). To account for potential taxonomic biases, we compared our results using species currently recognized by the Checklist committees of the American Ornithological Society (AOS; *n* = 210; S2 Table) to a second treatment consisting of more inclusive monophyletic groups representing either single species or species complexes (hereafter “lumped” species; *n* = 179; S2 Table). Taxonomic uncertainty arises where two currently recognized species are allopatric; thus, combining species complexes into single species should alleviate artifacts caused by uneven taxonomic splitting across species. The inclusion of both taxonomic treatments allowed us to assess if any of our results were caused by latitudinal biases in the frequency of paraphyletic species [26] or in species delimitation [27]. We found similar results in both taxonomies (Fig 3).

An alternative way to account for taxonomic bias, assuming that taxa that contain multiple species are older, is to condition on species age in the multivariate models and assess whether the correlation remains significant. We found that latitudinal midpoint still showed a negative (β = −0.001) and significant (*p* < 0.00003) correlation with phylogeographic structure, even when we corrected for species stem age. Additionally, we randomized the phylogeographic structure value in each species to produce a null distribution and compared univariate models examining latitude using randomized versus observed values. The association between latitude and phylogeographic structure was significantly different than the null expectation (*p* = 0; S2 Fig; S3 Table). Even though our sensitivity analyses cannot exhaustively account for all potential biases in species limits, we show that latitudinal variation in phylogeographic structure was robust to alternative taxonomies and species ages and significantly different from a null model.

### Most variables poorly explain phylogeographic variation

Despite sampling birds in diverse environments and with varied ecologies, these traits were often poor or inconsistent predictors of phylogeographic structure. From our measurements of museum specimens, we compiled data on species with a wide range of wing shapes (hand-wing index range: 4.873–64.327), body sizes (tarsus length range: 4.25–64.48 mm), and life history strategies, ranging from sedentary taxa to long-distance seasonal migrants (max = 7,833 km; S2 Table). We found that proxies for dispersal ability (hand-wing index), body sizes (tarsus length), and migratory distances were not significantly correlated with phylogeographic structure (S3 Table). Mean elevational preference, overall landscape ruggedness, and changes in climate since the Last Glacial Maximum were also not generally significant effects in our models, but many of the contemporary environmental variables that covary with latitude were (S3 Table). At the macroscale, phylogeographic diversity primarily varied along latitude, irrespective of the inclusion of other variables.

### Mechanisms potentially responsible for higher phylogeographic diversity in the tropics

Three primary processes might be responsible for higher phylogeographic diversity in the tropics: a higher rate of splitting of phylogeographic clusters, lower rate of extinction of phylogeographic clusters, or more time for phylogeographic structure to accrue in tropical species. Although most phylogeographic trees contain too few lineages to jointly estimate splitting and extinction rates within each species, they do contain information on the relative rate at which phylogeographic structure was formed and lost [28] that can be used to compare broad patterns across species. Diversification processes are more challenging to estimate from phylogeographic data of extant species than are the indices of phylogeographic diversity examined above, but latitudinal variation in these processes is of broad interest, and we address each process here.

#### Greater age of tropical species

**Fig 4:**
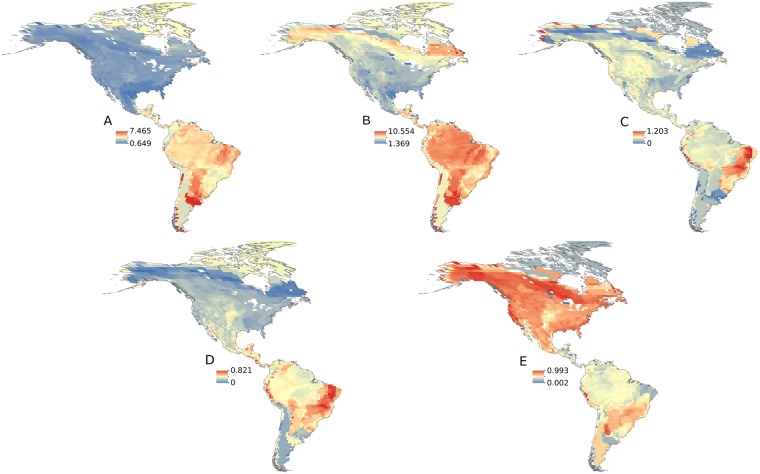
Heat maps show older tropical species, inconsistent support for higher splitting rates in the tropics, and greater lineage loss in temperate species. Shown are mean crown (A) and stem (B) ages in units of millions of years ago (Mya), mean crown (C) and stem (D) splitting rates, and lineage loss (E). Crown age is the time when extant mitochondrial DNA (mtDNA) haplotypes within each species coalesce. Stem age is the time when the mtDNA haplotypes coalesce with the species’ last common ancestor. Splitting rates were estimated using a pure-birth model. Lineage loss is a relative index, gauging the loss of lineages as determined from the standardized length of the stem branch (see Materials and methods). Warmer colors denote higher values.

Although latitude was a significant predictor of phylogeographic structure, after controlling for species age (see above), latitudinal differences in species age are still partly responsible for the association. Species age was strongly associated with latitude (crown age model; Adjusted R^2^: 0.406; *p* < 0.001), and heat maps of mean crown and stem ages show pronounced differences along the latitudinal gradient in which tropical species were older than those in the temperate region (Fig 4A & 4B; S1A & S1B Fig). We also found that species age was a strong predictor of phylogeographic structure across the various models we evaluated (stem age: ΔAICc: 27.488; crown age: ΔAICc: 69.072; S3 Table). These models explained approximately 35% to 47% (Adjusted R^2^: 0.349/0.467) of the variation in phylogeographic structure across species. The positive association between species age and amount of phylogeographic structure was robust to the threshold used to delimit the number of phylogeographic clusters, the uncertainty in species age, and alternative taxonomies (S3 Table) and was significantly different from a null model (*p* = 0; S2 Fig). In summary, we found that tropical species as currently delimited are older than temperate species, and older species have higher phylogeographic structure than younger species.

#### Higher tropical splitting rate

We estimated the rate at which phylogeographic structure formed, or the splitting rate, by fitting species age and the number of phylogeographic clusters within species to a pure-birth model (see Materials and methods). We found inconsistent support for faster splitting rates in tropical species. Heat maps show some evidence of higher mean splitting rates in tropical species in the estimates based on the stem age but not the crown age (Fig 4C & 4D; SD is shown in S1C & S1D Fig). Stem age, however, is less informative than crown age for inference of splitting rates [29] and may better reflect the time during which a lineage has persisted (see below) than the time over which it has diversified. A similar pattern was recovered with the multivariate models using splitting rates based on stem age (adjusted R^2^: 0.0746; *p* = 0.002; S3 Table), in contrast to those estimated from crown age (adjusted R^2^: 0.032; *p* = 0.066; S3 Table). Inconclusive support for latitudinal variation in splitting rates was further observed with comparisons of the empirical data to null models. Only stem splitting rates were significantly different than randomly produced values (stem splitting rates *p* = 0.04, S3 Fig; crown splitting rates *p* = 0.12, S4 Fig). The model results also varied among the alternative taxonomic datasets, as none of the splitting rate models using the lumped dataset were significant. Collectively, these data and analyses do not provide strong support that higher rates of splitting among tropical populations produced the observed latitudinal phylogeographic diversity gradient.

#### Lower tropical lineage loss rate

We calculated a proxy for lineage loss along the stem branch by measuring the standardized difference between stem and crown age (see Materials and methods). Long stem branches are expected in situations with high extinction rates, all else being equal [28]. Using this metric, we found that more lineage loss has occurred along the stem branch within temperate species than within tropical species (Fig 4E; SD Fig 1E). We found that lineage loss increased with latitude (β = 0.005; *p* < 0.00001; S3 Table), and that latitude was a significant predictor in the multivariate model (adjusted R^2^: 0.147; ΔAICc: 15.276; S3 Table) and was significantly different than a null model (*p* = 0; S5 Fig; S3 Table). Importantly, this metric should not be biased by taxonomy, as it is a standardized measure of branch length between two nodes. Higher lineage loss in temperate species was also observed in models that used climatic variables (the first principal component [PC1]) and net primary productivity, which were significant effects (ΔAICc: 14.225/7.415, respectively; S3 Table). Mirroring patterns of phylogeographic structure, tropical species show a broader range in the lineage loss index than temperate species, whose uniformly high lineage loss rates may underlie the latitudinal gradient in phylogeographic diversity.

## Discussion

Our comparative phylogeographic analysis of hundreds of New World bird species demonstrated that intraspecific genetic variation exhibits a pronounced latitudinal gradient. In comparison to temperate species, we found that tropical species are older and have accumulated and maintained more phylogeographic structure. These patterns are remarkably consistent with studies based on species-level data that show higher species richness [10], older taxa [30], and lower extinction in the tropics [11,31,32]. Although phylogeographic structure may not persist into the deeper evolutionary timescales examined in phylogenetic studies of species richness [33,34], the concordant patterns across timescales suggest that similar processes may be responsible in both cases. Overall, our results demonstrate that latitudinal diversity gradients are evident at shallow evolutionary timescales and that comparative phylogeographic data are useful for examining patterns of diversity at large geographic scales.

Higher climatic and environmental stability in the tropics has been implicated as a mechanism producing the latitudinal biodiversity gradient [10,35]. Pleistocene glacial—interglacial cycles had a global effect on species distributions and habitats [23], but these environmental effects were particularly profound in northern latitudes, where large ice sheets covered much of the terrain [36]. Our estimate of lineage loss, a standardized metric of stem branch length, showed the predicted latitudinal pattern of higher extinction in the temperate zone, where climatic conditions were most volatile. These findings reiterate that the lower genetic diversity in temperate species is due to long-term historical processes and not to human-modified changes to the landscape, as suggested by recent work [19]. Although long stem branches do not provide information on the magnitude of the number of lineages lost over time, their lengths are nevertheless indicative of relative levels of extinction and pruning [37,38]. Our results are consistent with studies directly estimating extinction at phylogenetic scales and among sister species, which found higher extinction rates in temperate birds [17,31]. Long stem branches in high-latitude species could alternatively reflect a failure of populations within northern species to diversify until recently, but this explanation seems unlikely, given the lack of an obvious biological reason (e.g., long-term environmental stability) for historical evolutionary stasis. A potential means by which temperate species could respond to both historical and seasonal climatic change is through long-distance migration, which is predicted to facilitate diversification [39]. However, our measurement of migration distance was not correlated with levels of phylogeographic structure. Low phylogeographic structure and high lineage loss, irrespective of dispersal ability or migratory behavior, are consistent with climatic instability, leaving a strong signature of lowered evolutionary persistence within temperate species.

We identified species age as another important mediator of the latitudinal gradient in phylogeographic structure. Previous work on birds occurring in lowland Neotropical rainforests suggested that this age—diversity association is linked to species ecologies that influence evolutionary persistence in the landscape, with more sedentary and poorly-dispersing species being older and containing deeper phylogeographic structure across their ranges [8]. Reduced dispersal ability has been linked more directly to divergence in regional studies focused on lowland Neotropical [4] and South Pacific bird faunas [40]. Our study includes a more exhaustive survey of Neotropical birds in terms of range-wide sampling and number of species, and we did indeed identify many tropical species with low dispersal abilities and high phylogeographic structuring. However, there was substantial noise around this relationship, and dispersal ability did not emerge as an important predictor of phylogeographic structure, after accounting for other factors. In addition, our novel comparisons of species across biomes show uniformly lower phylogeographic structure in temperate species, irrespective of dispersal ability. The lack of an association between dispersal ability as estimated from hand-wing index and phylogeographic structuring may be an indication that species ecologies do not have the same influence on structuring populations in the temperate region as in the tropics, that temperate species have more generalized niches that predispose them to dispersing [41], or that long-term climatic instability at high latitudes is the predominant factor that shapes levels of intraspecific genetic variation.

The strong association between species age and phylogeographic structure in our dataset reflects a clear historical influence on intraspecific variation, but other aspects of species history (e.g., ancestral origin) were not accounted for in our analysis. The historical biogeography of New World birds has focused on particular regions [e.g., 42] and macroscales [e.g., 43], but the colonization times in each region are not known for many lineages. Resolving when species colonized regions is necessary to clarify if diversity is attributable to how long a lineage has been in a region [e.g., 14] or if diversification is linked to expansion into novel environments [e.g., 44,45]. The impacts of long-term interactions among species on phylogeography are not known, but the evolution of a species’ phylogeographic history may be influenced by codistributed species through ecological interactions. In the tropical mountains of the Andes, elevational replacement of closely related taxa has been linked to intraspecific competition during the history of populations [46]. If the evolutionary outcome of ecological processes varies between the temperate zone and the tropics, then species interactions could play a role in shaping the latitudinal phylogeographic diversity gradient.

Although existing studies have found broadly concordant latitudinal patterns in diversity and evidence for greater evolutionary stability in the tropics, a general explanation for the link between diversification processes and latitudinal patterns has proven elusive. Studies have variously found that speciation or origination rates in lineages inhabiting the tropics are higher, lower, or similar to those of lineages in the temperate zone [17,33,47,48]. Differences obtained across these studies may be partially attributable to sampling different phylogenetic depths and scales [11], which range from sister species [e.g., 17], to taxonomic clades [e.g., 31], and complete phylogenies [48]. Differences in the temporal scale of studies may highlight the different processes that are at work over different timescales (e.g., divergence versus persistence) but may also reflect differences in our ability to model processes, such as extinction, in different types of data. At shallow timescales, we did not find evidence for accelerated intraspecific diversification in high latitudes, as observed in rates estimated from sister species divergence times [17] and subspecific diversity [16]. Reconciling these different stories will require not only improved data and models but also integrating the insights gained from different types of data collected over varying timescales.

Comparative analyses, such as ours, are reliant on accurately delimited species. Avian taxonomy is complicated by several sources of bias, including greater efforts in describing diversity in the temperate zone, a lack of sampling to clearly delimit taxa in the tropics, and/or inconsistent criteria for species delimitation. A major perceived bias is that tropical species harbor multiple species, whereas temperate species have been finely split [27,49]. We evaluated this issue by accounting for species age in our analyses and by using an alternative lumped taxonomy. We found the latitudinal phylogeographic diversity gradient to be robust to taxonomic treatment and the age of recognized species. However, if all phylogenetic species were elevated to full species, as some have proposed [49], species may no longer contain sufficient variation for comparative studies of intraspecific diversity. Under such a treatment, the patterns we observed in our data would reflect trends in recent speciation rather than intraspecific diversity, but they would still attest to the fact that broad patterns in diversity can form over very shallow timescales. We suggest that, when done carefully, comparative studies such as ours capture important biological patterns and processes, irrespective of taxonomic considerations.

The accuracy of phylogeographic metrics at capturing genetic differentiation depends on the genetic markers employed and the density of sampling. We focused on a coarse-scale metric that reflected deep phylogeographic structure (*sensu* [2]) because the degree of population-level sampling varied across species, and our objective was to assess broad comparative patterns. Ecological effects on genetic differentiation may be more pronounced at the allelic level and may require large SNP-based datasets with dense sampling of individuals to detect associations. Alternatively, our proxies for species ecology may not accurately reflect the ecological processes that influence genetic variation. Another confounding factor in our estimates was the impact of coalescent stochasticity on mtDNA gene trees. However, it is unlikely that the significant relationships we recovered were artifacts of coalescent stochasticity because we sampled a large number of species, our estimates of phylogeographic structure were conservative, and we evaluated our results against null models. Our species ages are presumably overestimates because the method we employed did not take into account species tree—gene tree discordance and ancestral effective population sizes [50]. We do not expect this estimation error to be highly biased across species differing in phylogeographic structure. These limitations can be improved with genome-scale data and dense sampling, but obtaining large and comparable multilocus datasets for hundreds of species will likely not be possible for years to come. Furthermore, although genome-scale estimates of phylogeographic metrics will improve parameter estimation [51], explaining the causes of among-species phylogeographic variation will remain a challenge.

Our study focused on data from a relatively well-known and well-sampled group of organisms, New World birds. Despite that, we found assembling a dataset of this size and completeness to be challenging. Among the least straightforward aspects of assembling these data were filtering erroneous data (e.g., taxon misidentification) and successfully extracting data and metadata from previous publications. As datasets grow, it will become particularly challenging to identify high-quality data. We opted for both manually checking data and trees with published results, along with automated processing that produced plots for additional data inspection. There may well be more signal in our data than we could extract in the broad analyses presented here, and it could provide further insight into the processes responsible for the observed latitudinal phylogeographic diversity gradient. For example, population expansions have been important in northern temperate species [52], but we did not examine evidence of genetic bottlenecks, extirpation, and selective sweeps. Mechanisms directly responsible for the older age of tropical species will also require further investigation to determine if population persistence is due to more stable climates, tropical species colonizing areas earlier than the temperate zone, and/or additional taxonomic bias. More sophisticated models, the addition of spatially explicit statistics of genetic diversity, and larger multilocus datasets could provide higher resolution to these processes and perhaps reveal additional predictors of broad-scale patterns of diversity.

In conclusion, phylogeographic data play a central role in elucidating the spatial and temporal dynamics of shallow evolutionary processes, and our study demonstrates that these processes are linked to broader biodiversity patterns. Species vary considerably in intraspecific diversity and the accumulation of this variability is time-dependent. Tropical species are older and harbor more phylogeographic structure, whereas temperate species are younger and have signatures of lineage loss, suggestive of pervasive impacts of environmental instability at high latitudes. Differential accumulation and persistence between tropical and temperate taxa may be correlated across phylogenetic scales, and this may produce gradients in both latitudinal phylogeographic structure and species diversity.

## Materials and methods

### Data collection

New World bird species are among the best-studied faunas at the phylogeographic level in sampling and diversity, and they occur across a broad environmental gradient that allows for comparative analysis at large spatial scales. We identified candidate phylogeographic datasets (S2 Table) and downloaded mtDNA sequence data from GenBank (S1 Data). We chose mtDNA because nuclear DNA was not available for the majority of species in our study. To standardize our collection approach and optimize comparability among species, we selected species for which geographical sampling was available and omitted populations/species that occurred on Caribbean islands or in the Old World, to focus our analysis on mainland North and South America. To obtain units for comparative analyses, we delineated species using the taxonomy of the North American (NACC) and South American (SACC) Checklist committees of the AOS [53,54]. We used two taxonomic delimitation treatments to account for biases caused by lumping and splitting of species. Our first treatment consisted of single species recognized by the AOS (*n* = 210; mean number individuals per species = 83.681; SD = 88.004). Some species had paraphyletic mtDNA gene trees, which is most likely attributed to taxonomic error [26]. These currently recognized biological species may not represent natural groups, particularly in tropical species [27], so we used a second taxonomic treatment in which closely related species were combined into species complexes and analyzed together. We combined all allopatric or parapatric populations and species that formed a monophyletic group for which we had range-wide genetic sampling. This alternative taxonomic treatment is referred to as the lumped dataset (*n* = 179; mean number individuals per species = 100.201; SD = 95.553).

### Estimating and visualizing phylogeographic variation

We estimated mtDNA gene trees for each species using BEAST v.1.7.5 [55]. Because the AOS species were nested within the lumped species, we built gene trees using the lumped data and extracted relevant values for each taxonomic treatment. We used published substitution rates to calibrate the mtDNA gene trees because there were no appropriate fossils. For the cytochrome *b* (cyt *b*) and cytochrome oxidase subunits I and II (COI and COII) genes, we used 0.0105 substitutions/site/million yr (s/s/my), a rate estimated for cyt *b* [56]. For the NADH dehydrogenase subunits II, III, and VI (ND2, ND3, ND6) and for ATPase6&8 genes, we used 0.0125 s/s/my, as estimated for ND2 [42]. Comparative analysis of whole mitochondrial genomes show that COI and COII evolve at a similar rate as cyt *b*, and that ND3 and ND6 evolve at a similar rate as ND2 [57]. Estimated rates of evolution for the control region are highly variable, ranging from 2% to 20% sequence divergence per million yr [58,59]. Because of the uncertainty surrounding the substitution rate of the control region, we opted for a conservative rate (0.0125 s/s/my) that was similar to that of the other, faster evolving mtDNA loci. For the uncorrelated lognormal relaxed clock mean (ucld.mean) parameter, we specified a lognormal distribution on the prior with the mean set to the above-mentioned mutation rates and a SD of 0.1. This dating approach allowed us to account for rate heterogeneity among genes and branches and for uncertainty around mean estimates. We used a coalescent-constant-size tree prior and the best-fit nucleotide substitution model as determined in MEGA6 [60], and we specified lognormal distributions on substitution model prior distributions. We ran each analysis for 50 million generations, sampling every 2,500 generations, performed multiple independent runs for validation, and assessed MCMC convergence and burn-in by examining ESS values and likelihood plots in Tracer v.1.5 [61]. For some datasets that did not achieve Effective Sample Size (ESS) values > 200 after 50 million generations, we added up to 50 million additional generations to ensure that the results were stable. For each focal species, we included the sister taxon (based on prior phylogenetic work) and extracted the mean and the 95% highest posterior density for stem (the time when the mtDNA haplotypes coalesce with the species’ last common ancestor) and crown (the time when all haplotypes in the species coalesce) age estimates for each species in units of millions of years ago (Mya). Some sister taxa did not share the same loci, and the resulting age estimates were incongruent, particularly for the stem age. To account for this discrepancy, when the data were available, we built multispecies alignments and ran BEAST analyses to obtain stem ages for multiple closely related taxa from a single tree. Mean stem (t = −2.72; SD = 2.70; *p* = 0.007) and crown ages (t = −2.51; SD = 1.66; *p* = 0.012) were older in the lumped dataset (stem age: mean = 4.81 Mya; SD = 2.95; crown age: mean = 2.23 Mya; SD = 1.78) than the dataset using currently recognized species (stem age: mean = 4.07 Mya; SD = 2.57; crown age: mean = 1.802 Mya; SD = 1.550).

Phylogeographic structure can be estimated via numerous means, including population genetic summary statistics [e.g., 62], assignment tests [e.g., 63] and tree-based approaches [e.g., 64]. For our study, we required a phylogeographic metric that could be estimated and compared across all species in our dataset that vary in terms of sampling. The multispecies coalescent provides an appropriate framework for delimiting genetic structure within species [65]. We used a Bayesian implementation of the General Mixed Yule Coalescent model (bGMYC)[24]. This model calculates the number of putative genetic species in a phylogeny by estimating the number of clusters in which the gene tree reflects intraspecific coalescent processes rather than interspecific processes. The bGMYC model provides a posterior probability that two tips in the phylogeny belong to the same genetic cluster, which can be used with a probability threshold to determine the number of clusters. We used the maximum clade credibility tree for each lumped species from BEAST for the bGMYC runs. We ran bGMYC for 250,000 generations using the single.phy function in R [66] and discarded the first 15,000 generations as burn-in. We ran each analysis multiple times for validation, and we assessed MCMC diagnostics by examining likelihood plots. We recorded the number of clusters per species using three different posterior probability thresholds (0.9, 0.8, and 0.7) to account for the uncertainty in delimited clusters. We recognize that finer-scale phylogeographic structure (e.g., significant F_ST_ values) was present in some species and that the approach we implemented cannot accommodate this level of genetic variation. Given this limitation, there may be interactions between species traits and genetic structuring that we lack sufficient resolution to infer. We found the mean number of phylogeographic units in a species was, as expected, significantly (t = −2.42; SD = 2.61; *p* = 0.016) higher in the lumped dataset (mean = 3.35; SD = 2.92) than the dataset using currently recognized AOS species (mean = 2.71; SD = 2.31).

We calculated a phylogeographic splitting rate under a pure-birth model, using the formula for stem age (equation 6) from Magallón and Sanderson [67] and the code in the R package laser [68]. We elected to not use a more complex model that estimates speciation and extinction rates from branching times because of the overall low number of nodes in the gene trees and their shallow depths. For example, in the dataset using currently recognized species (AOS species), the average number of phylogeographic clusters was less than three, with a mean age of less than 2 Mya. The Magallón and Sanderson [67] formula for diversification rates estimated from stem age assumes a starting diversity of one lineage, and the crown age formula assumes a starting diversity of two. There was no a priori reason to assume the starting number of phylogeographic units was two in a species, so we specified a starting diversity of one for the phylogeographic splitting rates estimated from both stem and crown ages. A comparison between the lumped dataset and the dataset using currently recognized AOS species showed that the splitting rates estimated from stem age were more similar (t = −1.23; SD = 0.250; *p* = 0.22; lumped stem rate: mean = 0.253; SD = 0.230; AOS stem rate: mean = 0.221; SD = 0.267) than the rates estimated from crown age (t = −1.82; SD = 0.641; *p* = 0.069; lumped crown rate: mean = 0.615; SD = 0.708; AOS crown rate: mean = 0.496; SD = 0.578).

In addition to calculating the degree of phylogeographic structure and splitting rate, we also calculated a stem branch index that served as a proxy for lineage loss in each species. We estimated this index by taking the difference between stem and crown age and standardizing the value by the stem age (Lineage Loss = [Stem Age−Crown Age]/Stem Age). We used mean and the 95% high and low values from highest posterior density to independently calculate lineage loss. The pruning of lineages by extinction will increase the stem branch index, but a failure to diversify could also leave a similar signature. To distinguish between these two processes, we used a mean branch length index (number of phylogeographic clusters/crown age) within the crown clade of each species as a reference for how long it takes diversification to begin, assuming that speciation occurred at a constant rate. Crown group branch length indices that are longer than stem branches could indicate that there has not been enough time for diversification to occur. We found that 15.2% of the species had crown group branch length indices longer than the stem branch lengths, which suggests these species may not have had enough time to diversity. However, phylogenetic generalized least-squares analysis (method details discussed below) recovered no strong latitudinal trends in the difference between these branch lengths (AOS species dataset: adjusted R^2^: −0.004; F-statistic = 0.269; *p* = 0.604; Lumped dataset: adjusted R^2^: −0.004; F-statistic = 0.242; *p* = 0.6234). The lack of a strong correlation between crown group branch length indices and stem branch lengths suggests that there is not a latitudinal bias in tropical or temperate species having less time to diversify.

We visualized phylogeographic data by projecting various metrics obtained from genetic data and our sampling strategy onto the 2-D plots of the New World. We downloaded digital range maps [69,70] and in ArcGIS 10.3 (ESRI Inc., Redlands, CA) converted breeding ranges to rasters with a cell size of 0.1 and then reclassified each raster cell in which the species was present to its number of phylogeographic clusters, splitting rate based on crown and stem age, lineage loss, species age, and to one to represent where the species occurred for the species richness map. All functions used to make heat maps were done in ArcGIS, and the processes described below were automated by using Python scripts with ArcGIS functions. We used the Cell Statistics function in Spatial Analysis Tools to summarize across the species' range maps and to generate per-cell values for mean, SD, and/or sum for the above listed variables. For the Cell Statistics function settings, we set the geographical extent and mask to mainland North and South America.

We also produced maps that visualize the proportion of total species sampled and the extent of sampling across each species range. We downloaded a global species-richness map of breeding birds constructed from the same digital range maps [69,70] used in our analyses, and estimated the proportion of species sampled per cell (Fig 1A) by dividing our species sampling map (Fig 1B) by a global species-richness layer [71] in Raster Calculator. To visualize sampling bias by producing sampling polygons (Fig 1C), we built sampling polygons for each species by obtaining latitude/longitude coordinates, converting coordinates to a shapefile (Split by Attribute add-in), converting the shapefile into a rectangle (Minimum Bounding Geometry function), and then clipping the rectangle to fall within each species range map (Clip function). We then produced polygons of unsampled areas by using the Erase function to identify the area in each species range not included in the sampling polygons. Sampling polygons were based on ten latitude-longitude coordinates that were compiled for each species from published records or georeferenced using descriptions of the sampling localities. For the lumped dataset, some lineages within the lumped species had fewer than ten samples. All points are plotted in S6 Fig Despite the large uncertainty surrounding some of these points, the coordinates, overall, provide coarse-scale resolution to how much of each species range was sampled. We summed the unsampled polygons (Cell Statistics function) and produced a heat map that shows areas that were undersampled (Fig 1C). We performed additional diagnostics on the association between the proportion of each species ranged sampled and phylogeographic structure (0.9 posterior probability threshold), range size, absolute latitudinal midpoint, and elevational preference (S7 Fig). Regression analyses indicated that there was only a significant association between the proportion of each species range sampled and phylogeographic structure (Adjusted R^2^: 0.059, *p* = 0.0002), but based on the other three plots, this bias was not associated with range size, absolute latitudinal midpoint, or elevational preference (S7 Fig). Finally, all maps were scaled to 110,000 km cells, to account for uncertainty in species range maps [72].

### Environmental data

We measured the environmental space each species inhabits by extracting precipitation, climate, and net primary productivity data from observational records. We also extracted data from layers measuring the difference between present-day and Last Glacial Maximum climatic conditions. Our objective was to compare phylogeographic metrics in species that inhabit more seasonal environments in the temperate regions with those that occur in less seasonal tropical environments. To do this, we used climatic layers that averaged across the annual cycle. These climatic data were not used to characterize the niches of species because some of the taxa (*n* = 52) in our dataset were migratory. Instead, the climatic data capture broad-scale habitat preferences (e.g., temperate broadleaf forests). We gathered 67,779 georeferenced observational records, representing all study taxa (mean = 83.2 records/species’ SD = 41.48; min/max = 1:147; S4 Table). We obtained records from eBird (May 2013 release), a real-time record of species distributions and abundances collected by amateur and professional ornithologists [73]. Prior to incorporation into the eBird database, all submitted observations are peer-reviewed by regional experts. Each record includes the start time, duration of data collection, and geographic distance covered. To minimize georeferencing inaccuracy while maximizing the number of localities per species, we included observations from all checklists that were less than 6 hr in duration and less than 5 km in distance traveled. For each species, we removed all duplicated localities and randomly selected 1000 records to which we applied a thinning algorithm such that no localities occurred within 1 km of each other, approximately the resolution of the climatic data grid cells. We further verified observational records against distributional maps [69,70].

For each locality record, we extracted elevation and 19 current climatic variables from the WorldClim database at a spatial resolution of 2.5 arc-seconds [74]. We also extracted net primary productivity for each record [75]. For each species, we estimated the range of climatic conditions inhabited by calculating the difference between the 95% high and low quantiles of each layer. The 95% range of climatic conditions acts as a proxy for breadth of habitat across a species range. We also incorporated climatic stability since the Last Glacial Maximum by measuring the per-cell difference between the 19 contemporary climatic layers and the corresponding paleoclimatic layers (MIROC: Model for Interdisciplinary Research on Climate) using the cell statistics function in Spatial Analysis Tools in ArcGIS. Using the eBird observational records, we extracted the cell values from each of these climatic stability layers. To reduce the dimensionality of the climatic niche estimates, we conducted a principal components analysis of the contemporary climatic variables, climatic stability variables, and elevation using the prccomp function in R [66]. We used the Kaiser Criterion (Eigenvalues greater than one) to reduce the number of components, and we retained principal components one through four. We calculated mean and standard error for the principal components for each species. For downstream analyses, we used either the first principal component (PC1), which explained 49% of the climatic variation across species (S5 Table), or a combination of annual mean temperature (BIO1), seasonality temperature (BIO4), annual mean precipitation (BIO12), and precipitation seasonality (BIO15).

For each currently recognized species, we determined the range size, maximum and minimum latitude, latitudinal range, landscape ruggedness, and midpoint of occurrence using digital range maps [69,70]. We projected the range map for each species with a lambert azimuthal equal area projection. All of the spatial variables we collected were from the resident distribution of each species or, in the case of migratory species, the breeding distribution. We estimated the area of each range and sampling polygon in km^2^ and calculated the proportion of range sampled by dividing the sampling polygon area by the range size. For migratory species, we calculated migratory distance as the difference between the breeding and wintering latitudinal midpoints of each species. For sedentary species, we specified migratory distance as zero. For species in the lumped dataset that consisted of more than one currently recognized species, we merged the range maps of these species and calculated the same metrics as above. We performed projections and calculations using the R packages maptools [76], raster [77], and rgdal [78]. To measure the topographic variability across species ranges, we used a modified Melton index [79]—(Elevation_max_−Elevation_min_)/log (range size)—that included a log-converted range size instead of a square root conversion, in order to account for the large variance in range sizes across species. We generated 250 random points per polygon in each species distribution, and we extracted the elevation at each point to estimate maximum and minimum elevation.

### Morphological data

We recorded wing length (WL), secondary length (SL), and tarsus length from vouchered specimens deposited at the American Museum of Natural History and the Museum of Natural Science at Louisiana State University (S6 Table). We measured five male specimens in adult plumage per species, and for migratory species, we only included individuals collected during breeding months. We selected males because females may show greater variation in mass during the breeding season than males [80]. The shape of a bird’s wing influences its flight capabilities and serves as a proxy for dispersal ability. Birds with long, narrow wings are more capable of long-distance flight than species with rounder, short wings [81]. We calculated a proxy for dispersal ability using the wing measurements (hand-wing index = 100 x (WL − SL)/SL), a metric that is positively correlated with dispersal ability [82]. We used tarsus length as a proxy for body size [83], as these are positively correlated for most species in our dataset with the exception of parrots (Order: Psittaciformes), which have relatively small tarsi given their body size.

### Comparative analyses

The biogeographical distributions of birds, including the species in our dataset, are nonrandom, with entire clades distributed only in the temperate or the tropical region. To account for this potential phylogenetic effect on patterns of latitudinal variation, as well as uneven geographical sampling among different groups of birds and the nonindependence of species trait data, we used PGLS [83] analysis. We tested whether variables were significantly correlated with different metrics of phylogeographic variation (phylogeographic structure, splitting rates, and lineage loss) by fitting data to a condensed set of multivariate models. The purpose of the multivariate modeling was to determine how much of the variation in the phylogeographic metrics could be explained by the predictor variables and to determine the relative importance of each of the variables.

We independently examined four classes of response variables reflecting the phylogeographic history of species from the AOS (*n* = 210) and lumped (*n* = 179) datasets: (1) the degree of phylogeographic structuring within species, as determined by the number of bGMYC species clusters; (2) species age, as determined by crown and stem ages; (3) the rate at which diversification occurs within species or splitting rate, as determined by the phylogeographic diversification rate estimated from stem and crown species ages; and (4) lineage loss, as determined by the standardized length of time between the stem and crown nodes. To account for uncertainty in parameter estimates, we independently modeled metrics (phylogeographic structure and splitting rates) using three clustering threshold values (0.9, 0.8, 0.7). We treated predictor variables as fixed effects in the models. To reduce the residual variance in the models we square root converted the following variables in the PGLS analysis—number of phylogeographic units, species age, range size, migratory distance, hand-wing index, and sample size. To account for the phylogenetic nonindependence of the species trait data, we used the Jetz et al. [48] tree, built using the Hackett et al. [85] phylogeny as a backbone. We downloaded 1,000 trees from birdtree.org (Hackett All Species option; January 2017), and we built a maximum clade credibility (MCC) tree using the pseudoposterior distribution of trees in Tree Annotator [55]. Four species in our dataset represent recent taxonomic changes and were not included in the Jetz et al. [48] tree. For these taxa, we grafted species onto the phylogeny to their sister taxon using the add.tip function in the ape package [86] in R [66]. All models are available in S3 Table. We used 100 subsampled trees for the multivariate models and the MCC tree for univariate tests.

Using the PGLS function in the R [66] caper package [84], we fit data to multivariate models. This function models phylogenetic signal in the data using the parameters lambda, kappa, and delta. We optimized the value for lambda using maximum likelihood, and we kept the default values for kappa (1.0) and delta (1.0). We assessed whether the empirical response variables were significantly different from a random sample of values generated using the same mean, SD, and distribution type of the empirical data. For all response variables, we used truncated lognormal distributions, except for lineage loss, for which we used a truncated normal distribution instead. Both functions are part of the EnvStats R package [87]. From these distributions, we produced random values of response variables (phylogeographic structure, species age, splitting rates, and lineage loss) for each species 100 times. We then ran univariate PGLS models, recorded the adjusted R^2^ values for each model, and then compared the proportion of simulated R^2^ values above the empirical R^2^ value. If <5% of the simulated values were greater than the empirical R^2^ value, we concluded that the empirical species trait values were not generated by this random process. We plotted null models for phylogeographic structure (S2 Fig), splitting rate using stem age (S3 Fig), splitting rate using crown age (S4 Fig), and the lineage loss index (S5 Fig).

For multivariate models, we estimated AICc scores with a correction for sample size for a full model with all variables and the AICc score for each model without each of the predictor variables. We assessed the relative importance of each variable by calculating ΔAICc = AICc_a_ − AICc_f_, where ΔAICc is the change in AICc between the model without a particular predictor variable (AICc_a_) and the full model (AICc_f_). Models with a ΔAICc > 2 are deemed to be significantly less likely than the full model, and the removed variable is considered important. We report model output from the median AICc score of the 100 full models, based on each of the 100 different trees. We then used the tree that produced the median AICc score for the full model to report the output for the alternative models. Because the environmental variables in our dataset are correlated, we ran different sets of multivariate models with uncorrelated variables. Each set of models assessing variable importance included only one of following: (1) latitudinal midpoint; (2) net primary productivity; (3) mean temperature and precipitation, and temperature and precipitation seasonality; (4) bioclimatic PC1; (5) climatic instability since the Last Glacial Maximum; and (6) range in mean temperature and precipitation, temperature and precipitation seasonality, and elevational preference. We report AICc weights in order to show the relative stability of similar models using different treatments. We did not perform model averaging because the large numbers of variables and models would lead to data dredging, and interpreting the biological significance of these models would be difficult.

Our predictions for the influence of variables on phylogeographic metrics are shown in S1 Table. To briefly summarize, we expect that phylogeographic structure will be higher in older species that persist in the landscape [e.g., 8], inhabit areas that were more climatically stable between glacial—interglacial periods [e.g., 88], are distributed in areas with more energy [e.g., 28], inhabit more topographically complex areas [e.g., 89] or broader environmental conditions, have lower dispersal abilities [e.g., 4,41], and have larger geographical ranges. We expect similar associations with splitting rates and species ages. The climatic stability of an area has been suggested to both increase and decrease diversification rates [16,17,28]. Splitting rates may asymptotically increase with range size as species fill up geographical space. Alternatively, species ranges may be dynamic and decoupled from their rate of diversification. The geographic distance species migrate may facilitate diversification by allowing species to rapidly colonize new environments [39], or migratory behavior may alternatively inhibit diversification by limiting isolation among populations via gene flow [e.g., 90]. We predict that lineage loss will be higher in temperate latitudes, species with smaller habitat breadth, areas with greater historical climatic instability, and species with higher dispersal abilities, longer migratory distances, and smaller ranges.

### Additional trait data

We also mined foraging guilds (Fig 1D) and body sizes (Fig 1E) for sampled and unsampled New World bird species from the EltonTraits database [25]. Because our dataset, while large for a comparative phylogeographic study, includes only up to ~10% (Fig 1A; South American tropics) to ~30% (Fig 1A; temperate North America) of the total diversity, we compared the two above mentioned traits to unsampled species.

## Supporting information

S1 Fig Heat maps showing standard deviation for corresponding maps in Fig 4. Shown are crown (A) and stem (B) ages and mean crown (C) and stem (D) splitting rates, and lineage loss (E) standard deviation. Crown age is the time in which extant mtDNA haplotypes within each species coalesce. Stem age is the time of when the mtDNA haplotypes coalesce with the species’ last common ancestor. Splitting rates were estimated using a pure-birth model. Lineage loss is a relative index gauging the loss of lineages as determined from the standardized length of the stem branch, see Materials and methods. Warmer colors denote higher values.
(PDF)

S2 Fig Histograms of null models comparing adjusted R^2^ values from randomized and empirical phylogeographic structure values. Red lines are empirical values and black lines are the 95% quantile threshold of the R2 values from models using randomized values. The x-axis shows R2 values for the predictor variable used in each univariate comparison. Additional model output and underlying data are can be found in S3 and S7 Tables.
(PDF)

S3 Fig Histograms of null models comparing adjusted R^2^ values from randomized and empirical stem splitting rates. Red lines are empirical values and black lines are the 95% quantile threshold of the R^2^ values from models using randomized values. The x-axis shows R^2^ values for the predictor variable used in each univariate comparison. Additional model output and underlying data are can be found in S3 and S7 Tables.
(PDF)

S4 Fig Histograms of null models comparing adjusted R^2^ values from randomized and empirical crown splitting rates. Red lines are empirical values and black lines are the 95% quantile threshold of the R2 values from models using randomized values. The x-axis shows R2 values for the predictor variable used in each univariate comparison. Additional model output and underlying data are can be found in S3 and S7 Tables.
(PDF)

S5 Fig Histograms of null models comparing adjusted R^2^ values from randomized and empirical lineage loss indices. Red lines are empirical values and black lines are the 95% quantile threshold of the R^2^ values from models using randomized values. The x-axis shows R^2^ values for the predictor variable used in each univariate comparison. Additional model output and underlying data are can be found in S3 and S7 Tables.
(PDF)

S6 Fig Geographical coordinates for species used to produce sampling polygons. Ten latitude-longitude coordinates were compiled for each species from published records or georeferenced using descriptions of the sampling localities. For the lumped dataset some lineages within the lumped species had less than 10 samples.
(PDF)

S7 Fig Scatter plots with regression lines showing the relationship between sampling and various variables. On the y-axis of each plot is the proportion of range size sampled versus phylogeographic structure as determined by the number of bGMYC clusters using a 0.9 threshold (top left), absolute latitudinal midpoint (top right), range size km2 (bottom left), and mean elevational (m) occurrence (bottom right). Summary of regression for each plot is as follows: Phylogeographic Structure: Adjusted R^2^: 0.059, p-value: 0.0002; Absolute Latitudinal Midpoint: Adjusted R^2^: 0.015, p-value: 0.043; Range Size: Adjusted R^2^: 0.004, p-value: 0.174; Elevation: Adjusted R^2^: -0.004, p-value: 0.74. Blue line and grey shading are the regression line and the 95% CI of the slope, respectively. The underlying data can be found in S2 Table.
(PDF)

S1 Table Predictions of variable influence on phylogeographic metrics. The expected directionality of the correlation are shown: positive (+) or negative (-). N/A indicate there is no clear indication of how the variables will interact.
(DOCX)

S2 Table Spreadsheet containing taxon list and species data values. (XLSX)

S3 Table Spreadsheet containing PGLS models and output. (XLSX)

S4 Table Text file containing latitude and longitude coordinates mined from eBird. These coordinates were used to extract environmental data for each species.
(TXT)

S5 Table Output from principal components analysis of 19 climatic variables. Shown are factor loadings, eigenvalues, and the percentage of the variation explained for each of the first four PC axes.
(DOCX)

S6 Table Spreadsheet containing taxon list and morphological data. (XLSX)

S7 Table Spreadsheet containing R^2^ values from univariate null models. These data values were used to produce the histograms in S2–S5 Figs.
(XLSX)

S1 Data Zip file containing mitochondrial DNA sequences alignments as nexus files for the species listed in S2 Table. (ZIP)

## Abbreviations

AICc: Akaike information criterion
AOS: the American Ornithological Society
mtDNA: mitochondrial DNA
Mya: millions of years ago
PC1: the first principal component
PGLS: phylogenetic generalized least-squares
SD: standard deviation
