# Correction: A latitudinal phylogeographic diversity gradient in birds

**DOI:** 10.1371/journal.pbio.1002610

**Published:** 2017-07-14

**Authors:** Brian Tilston Smith, Glenn F. Seeholzer, Michael G. Harvey, Andrés M. Cuervo, Robb T. Brumfield

Within the Materials and Methods, there is an error in the hand-wing index equation in the section titled "Morphological data." The published article incorrectly refers to the secondary length (SL) when intending to refer to the wing length (WL) in the denominator. Please view the complete, correct equation here: hand-wing index = 100 x (WL − SL)/WL
