# Supplementary figures and images for: A latitudinal phylogeographic diversity gradient in birds
Source: PLoS Biol. 2017 Apr 13;15(4):e2001073. doi: 10.1371/journal.pbio.2001073 (PMC5390966; doi:10.1371/journal.pbio.2001073)

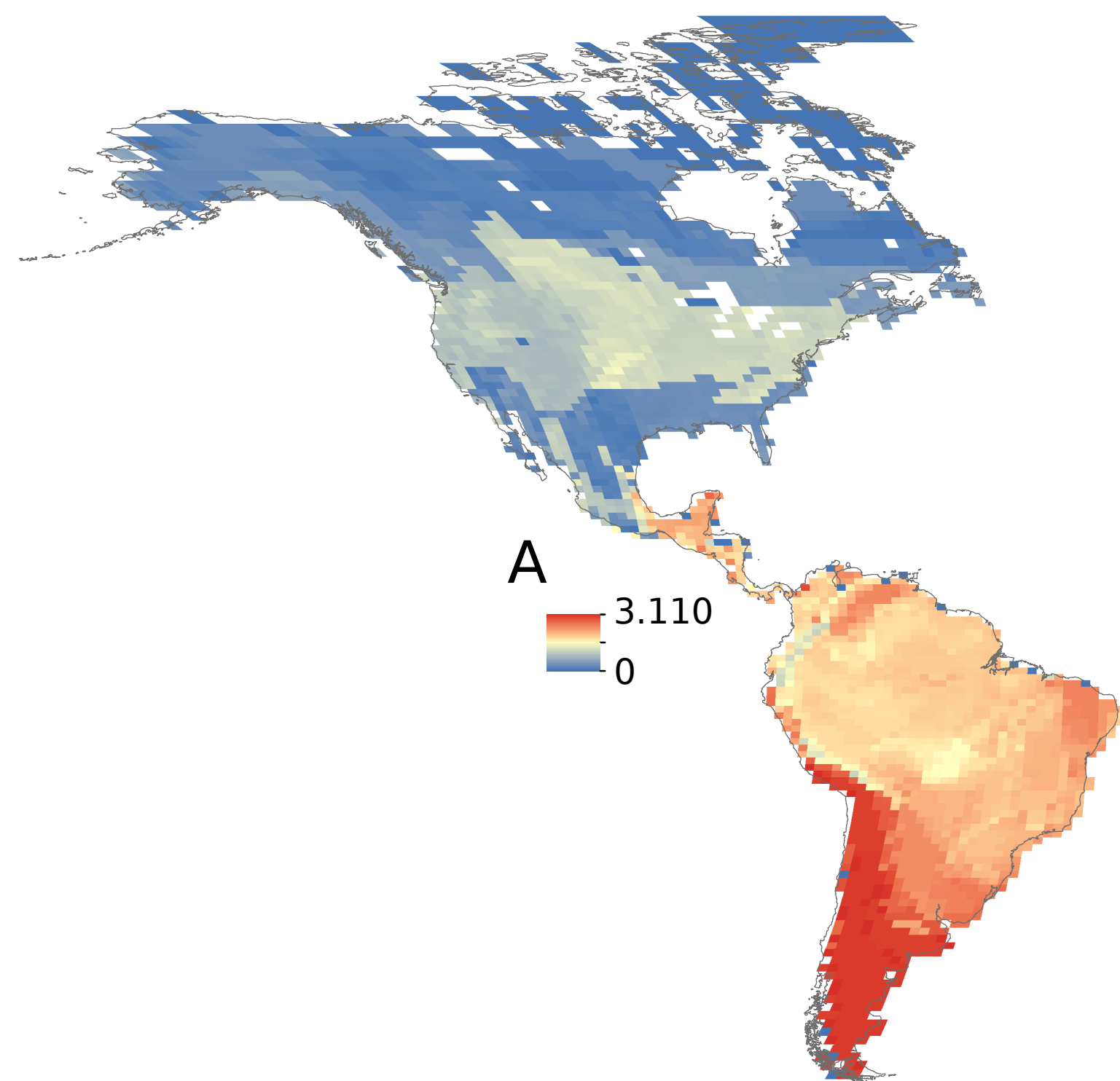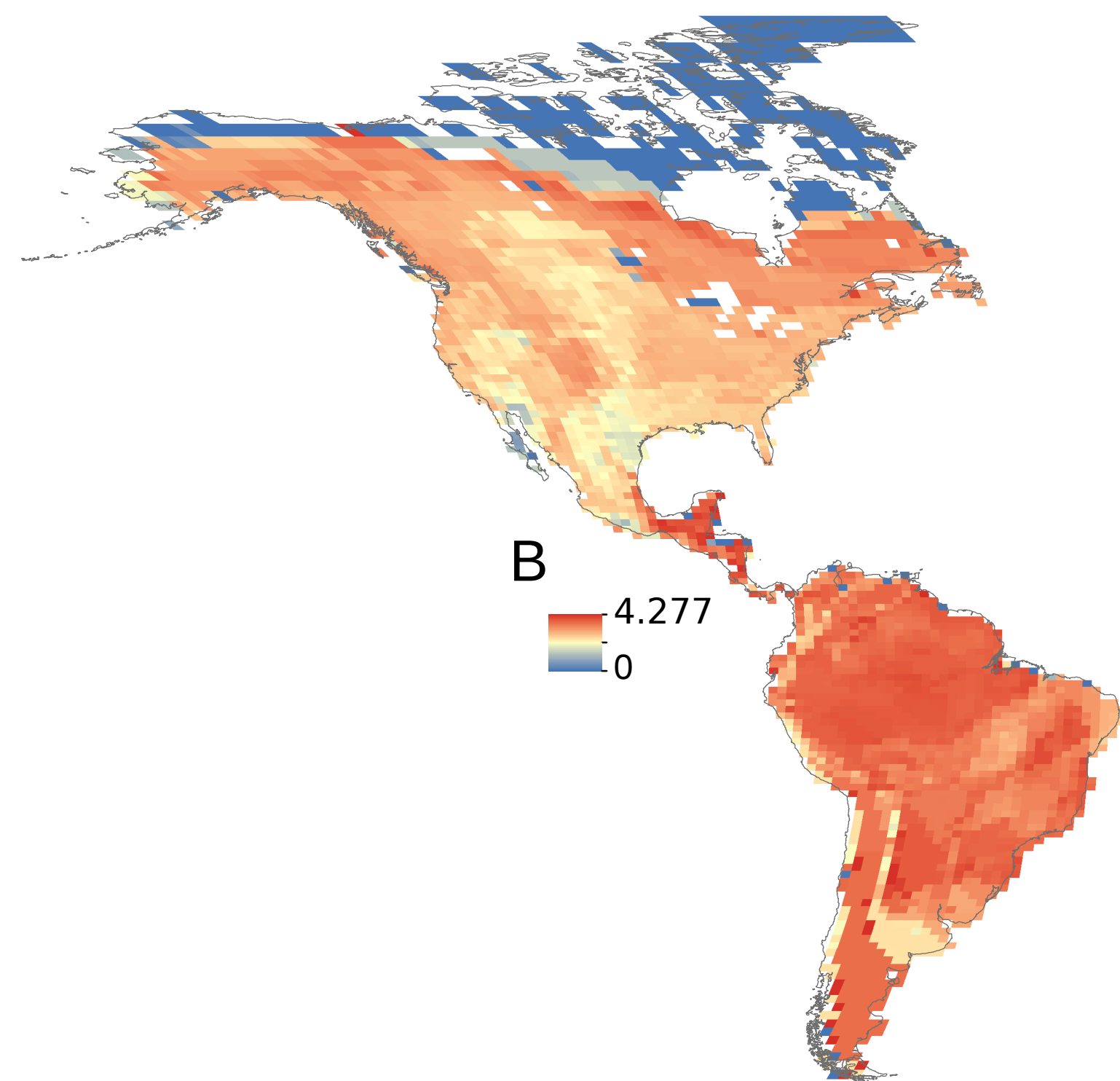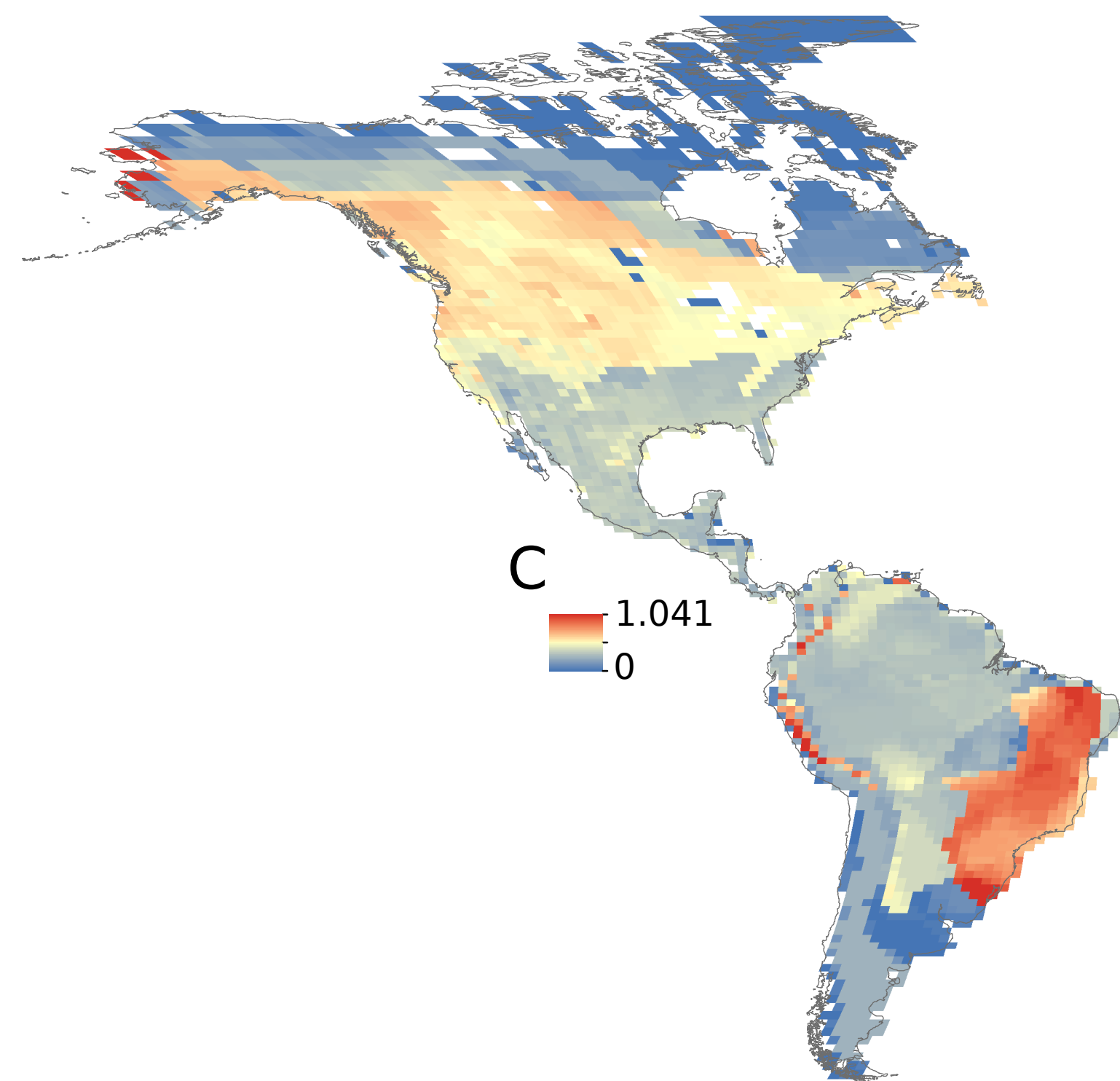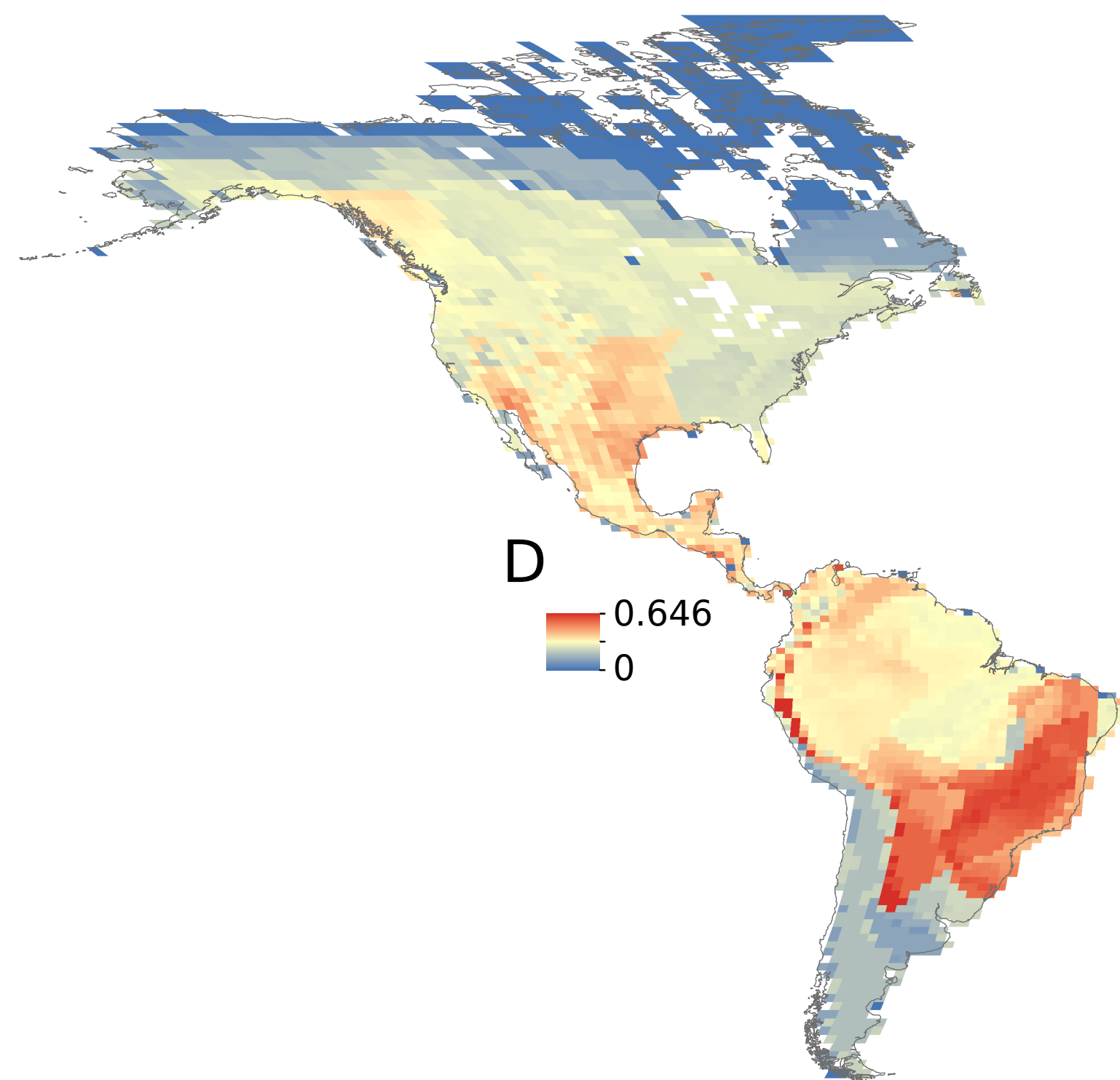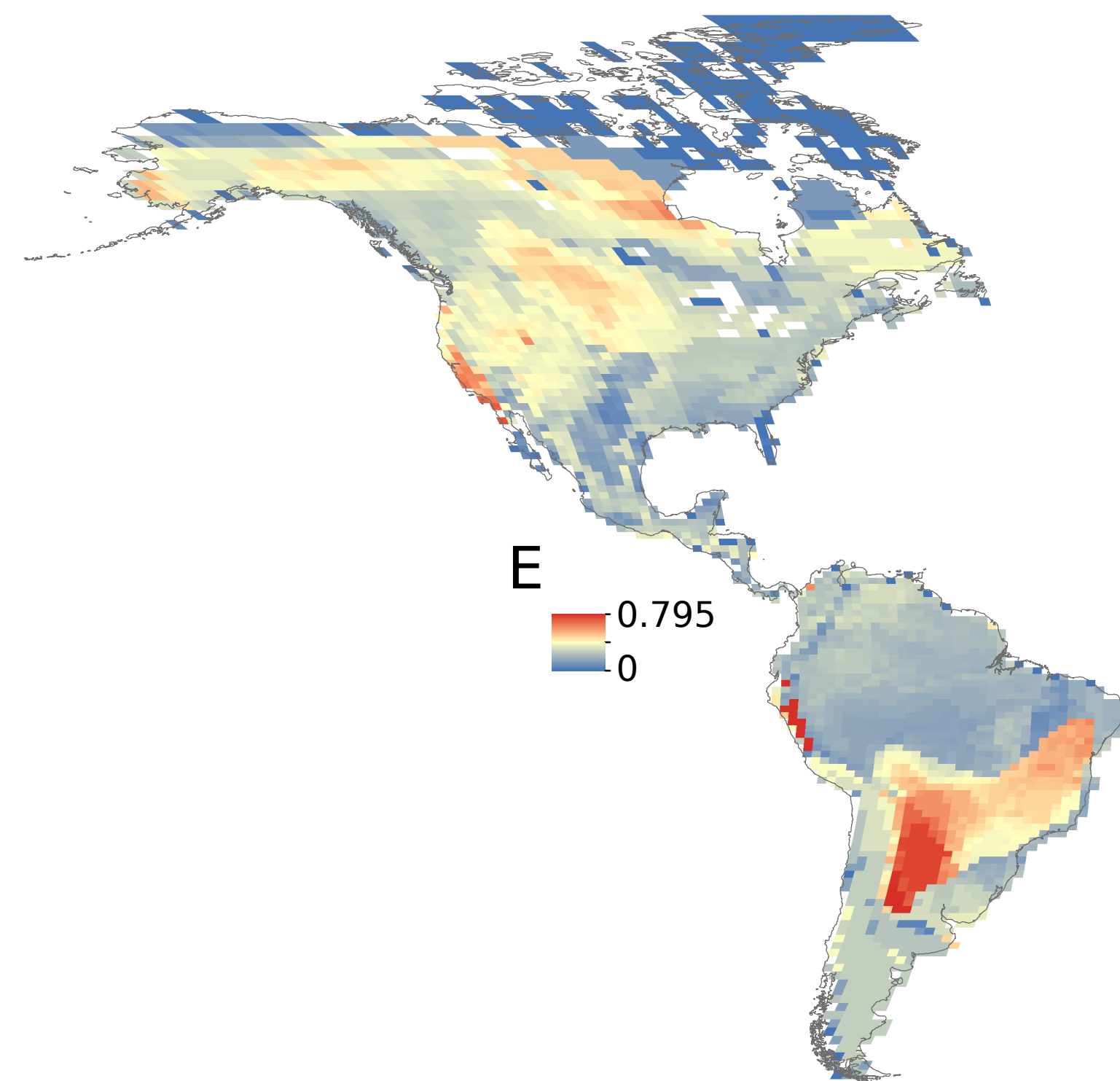

Supplement: S1 Fig — Shown are crown (A) and stem (B) ages and mean crown (C) and stem (D) splitting rates, and lineage loss (E) standard deviation. Crown age is the time in which extant mtDNA haplotypes within each species coalesce. Stem age is the time of when the mtDNA haplotypes coalesce with the species’ last common ancestor. Splitting rates were estimated using a pure-birth model. Lineage loss is a relative index gauging the loss of lineages as determined from the standardized length of the stem branch, see Materials and methods. Warmer colors denote higher values. (PDF) [file pbio.2001073.s001.pdf]

# Phylogeographic Structure

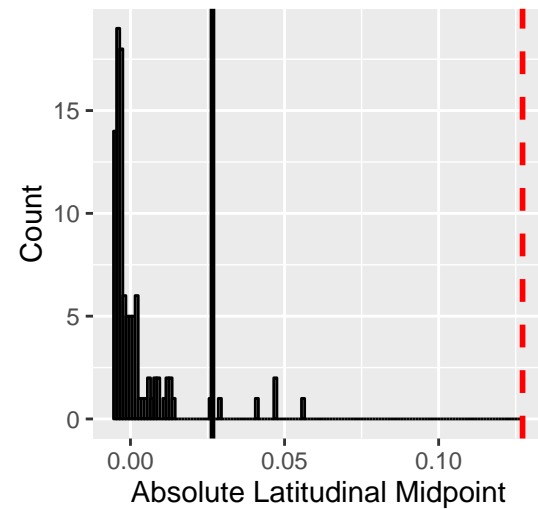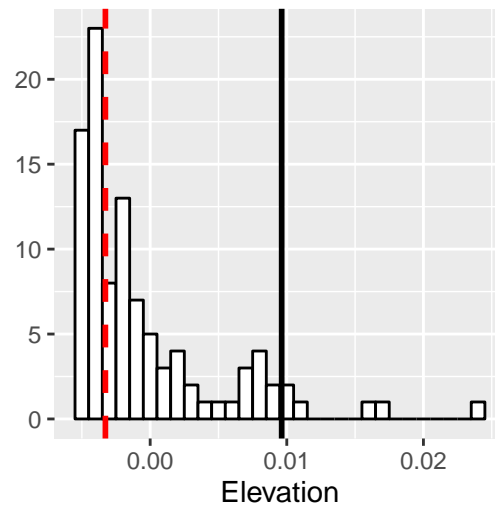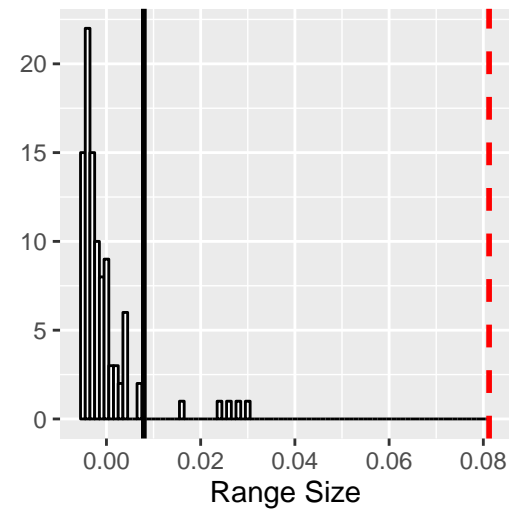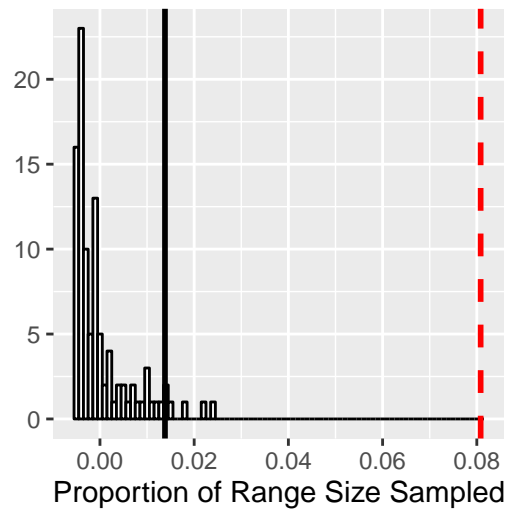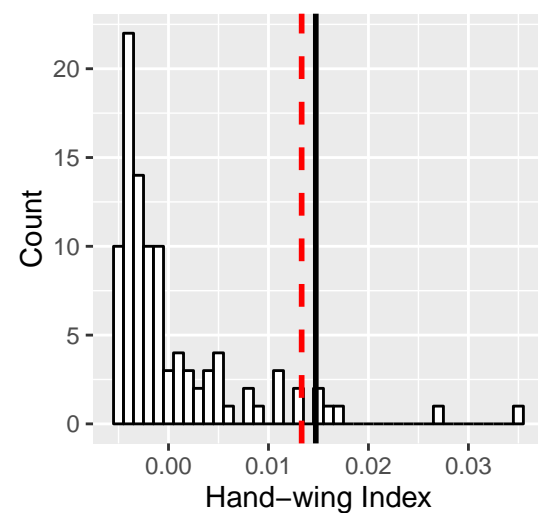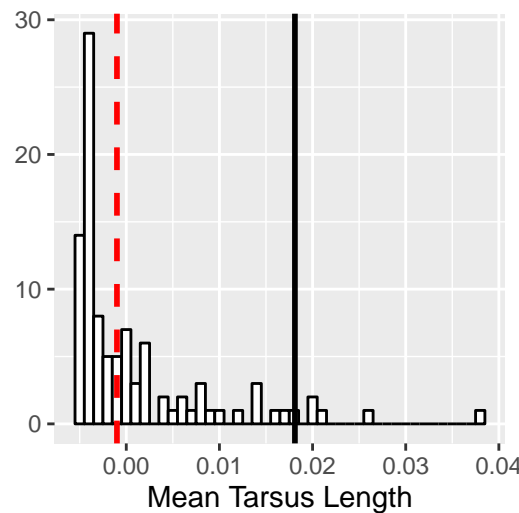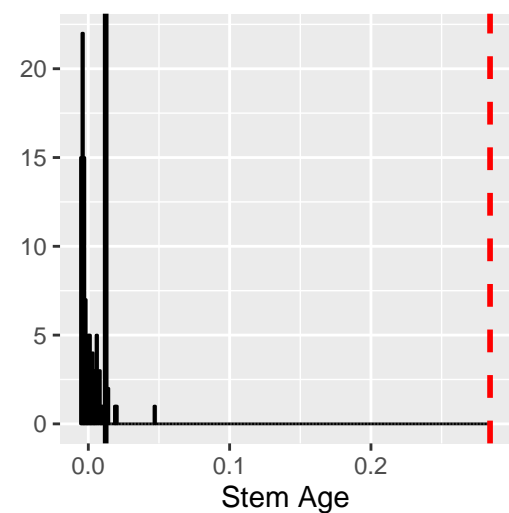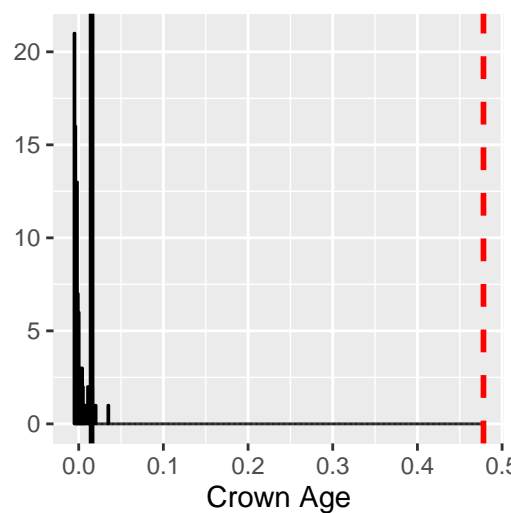

Supplement: S2 Fig — Red lines are empirical values and black lines are the 95% quantile threshold of the R2 values from models using randomized values. The x-axis shows R2 values for the predictor variable used in each univariate comparison. Additional model output and underlying data are can be found in S3 and S7 Tables. (PDF) [file pbio.2001073.s002.pdf]

## Stem Splitting Rate

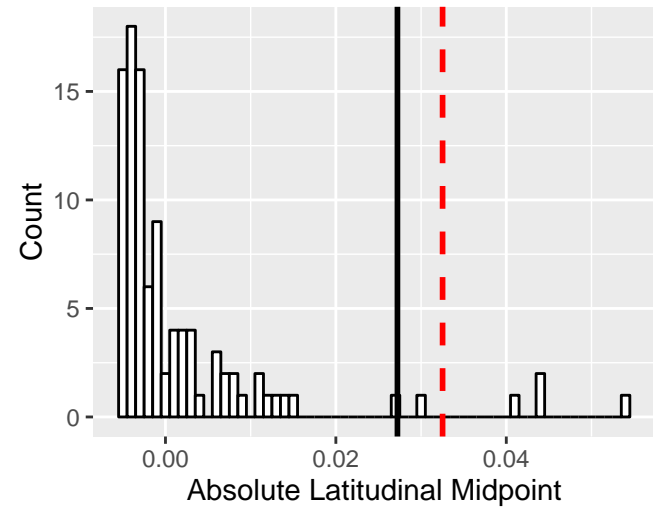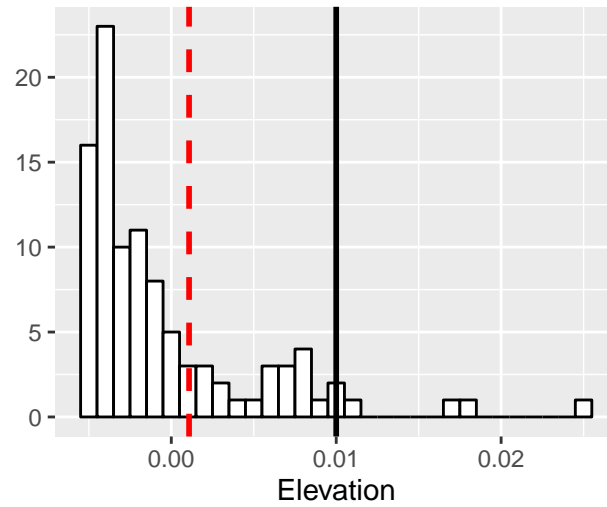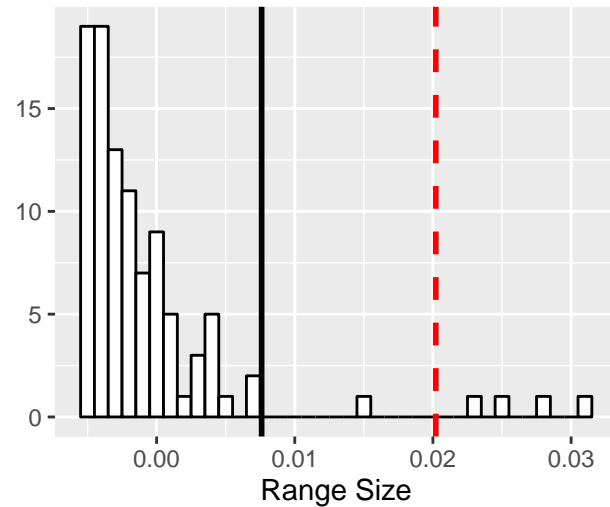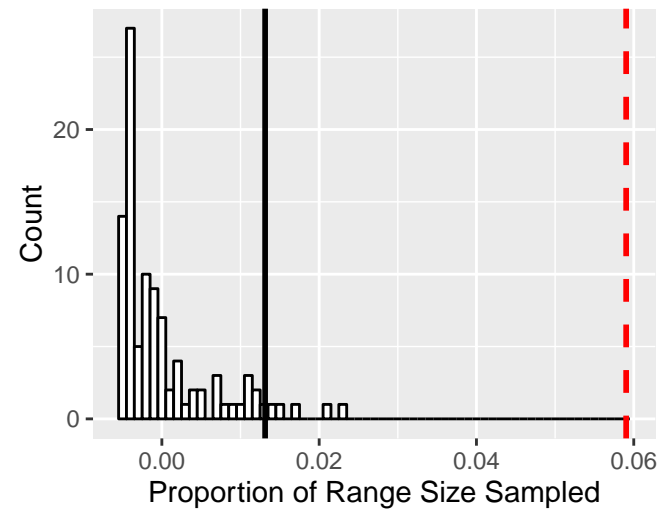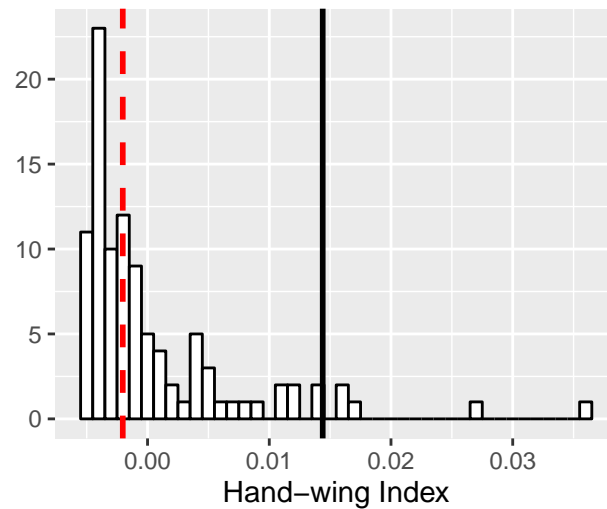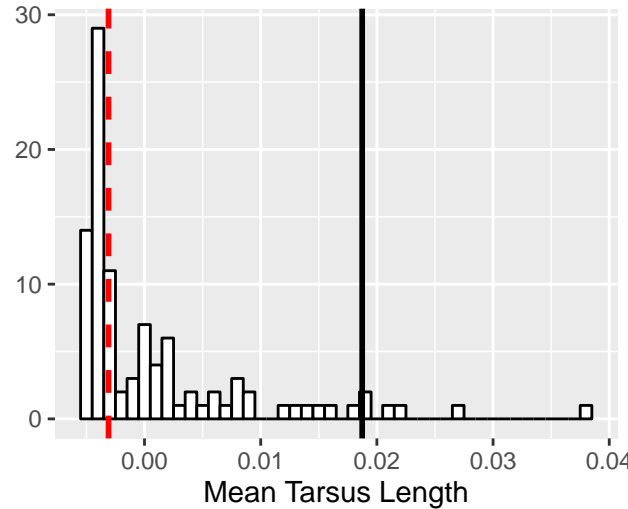

Supplement: S3 Fig — Red lines are empirical values and black lines are the 95% quantile threshold of the R2 values from models using randomized values. The x-axis shows R2 values for the predictor variable used in each univariate comparison. Additional model output and underlying data are can be found in S3 and S7 Tables. (PDF) [file pbio.2001073.s003.pdf]

## Crown Splitting Rate

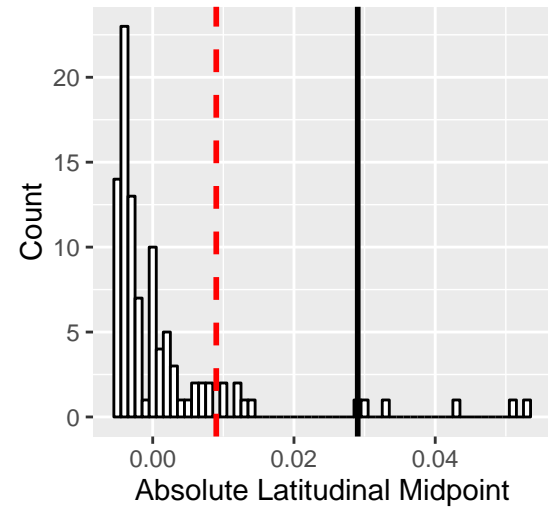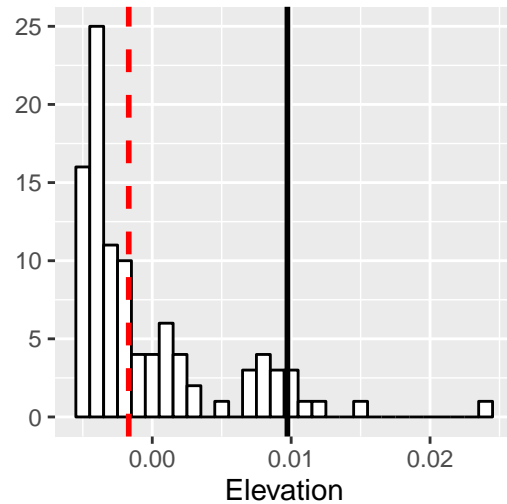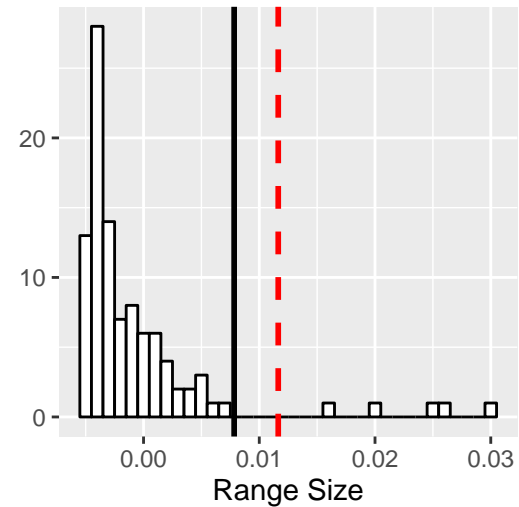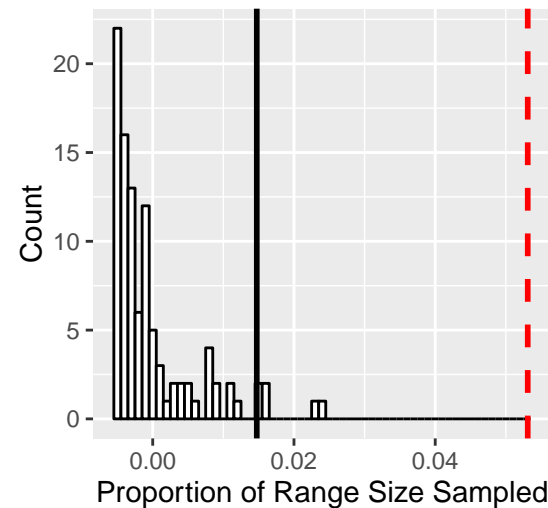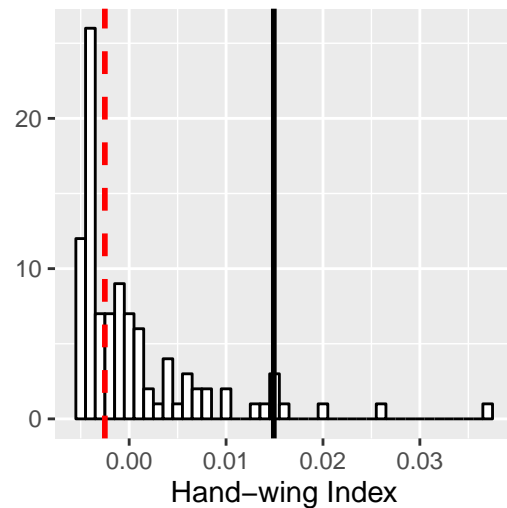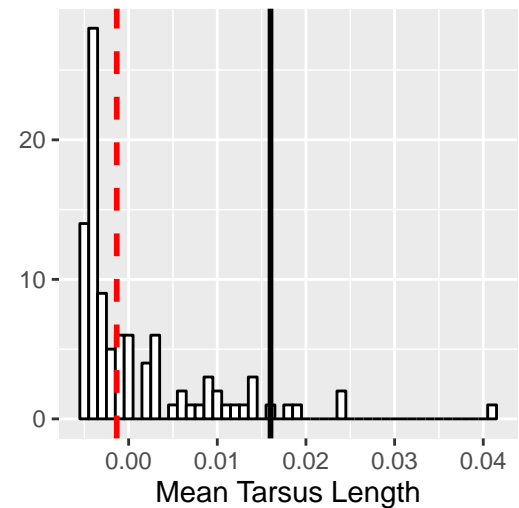

Supplement: S4 Fig — Red lines are empirical values and black lines are the 95% quantile threshold of the R2 values from models using randomized values. The x-axis shows R2 values for the predictor variable used in each univariate comparison. Additional model output and underlying data are can be found in S3 and S7 Tables. (PDF) [file pbio.2001073.s004.pdf]

# Lineage Loss Index

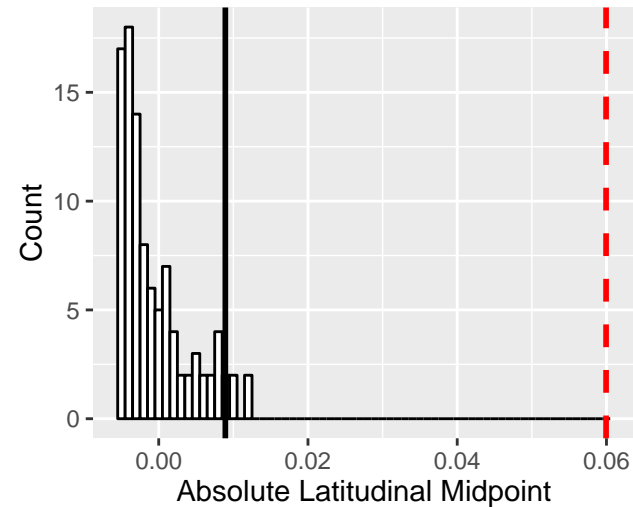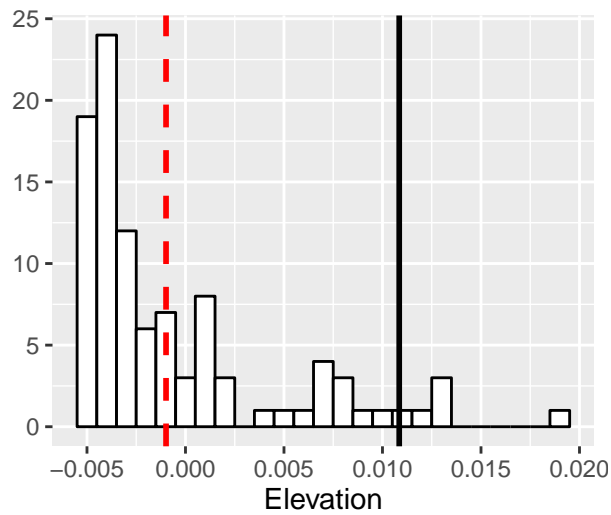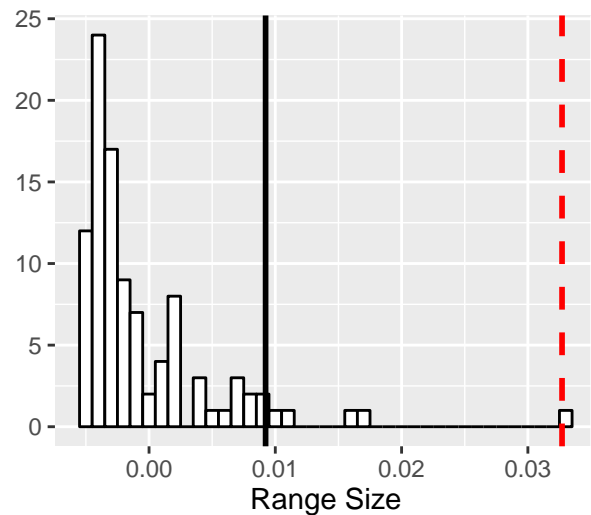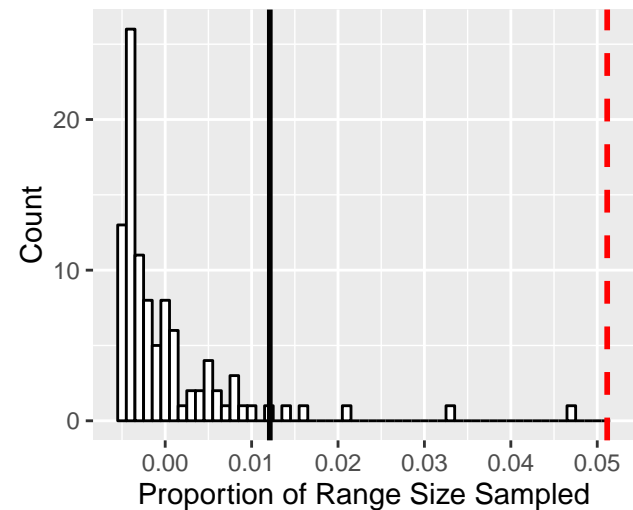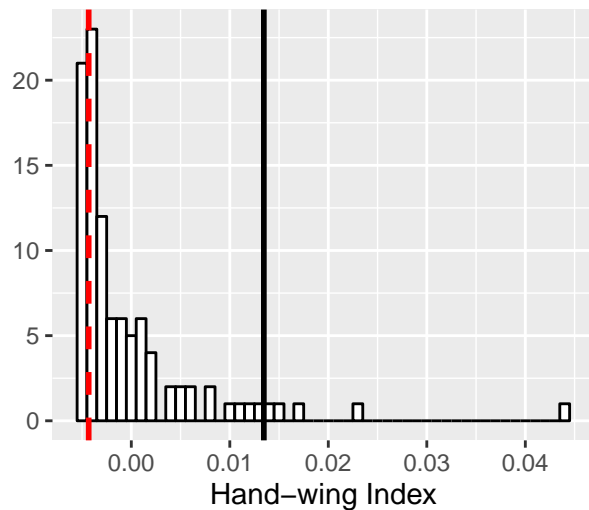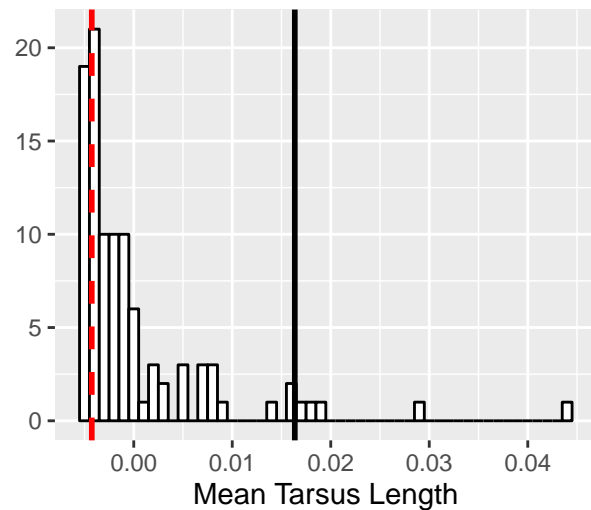

Supplement: S5 Fig — Red lines are empirical values and black lines are the 95% quantile threshold of the R2 values from models using randomized values. The x-axis shows R2 values for the predictor variable used in each univariate comparison. Additional model output and underlying data are can be found in S3 and S7 Tables. (PDF) [file pbio.2001073.s005.pdf]

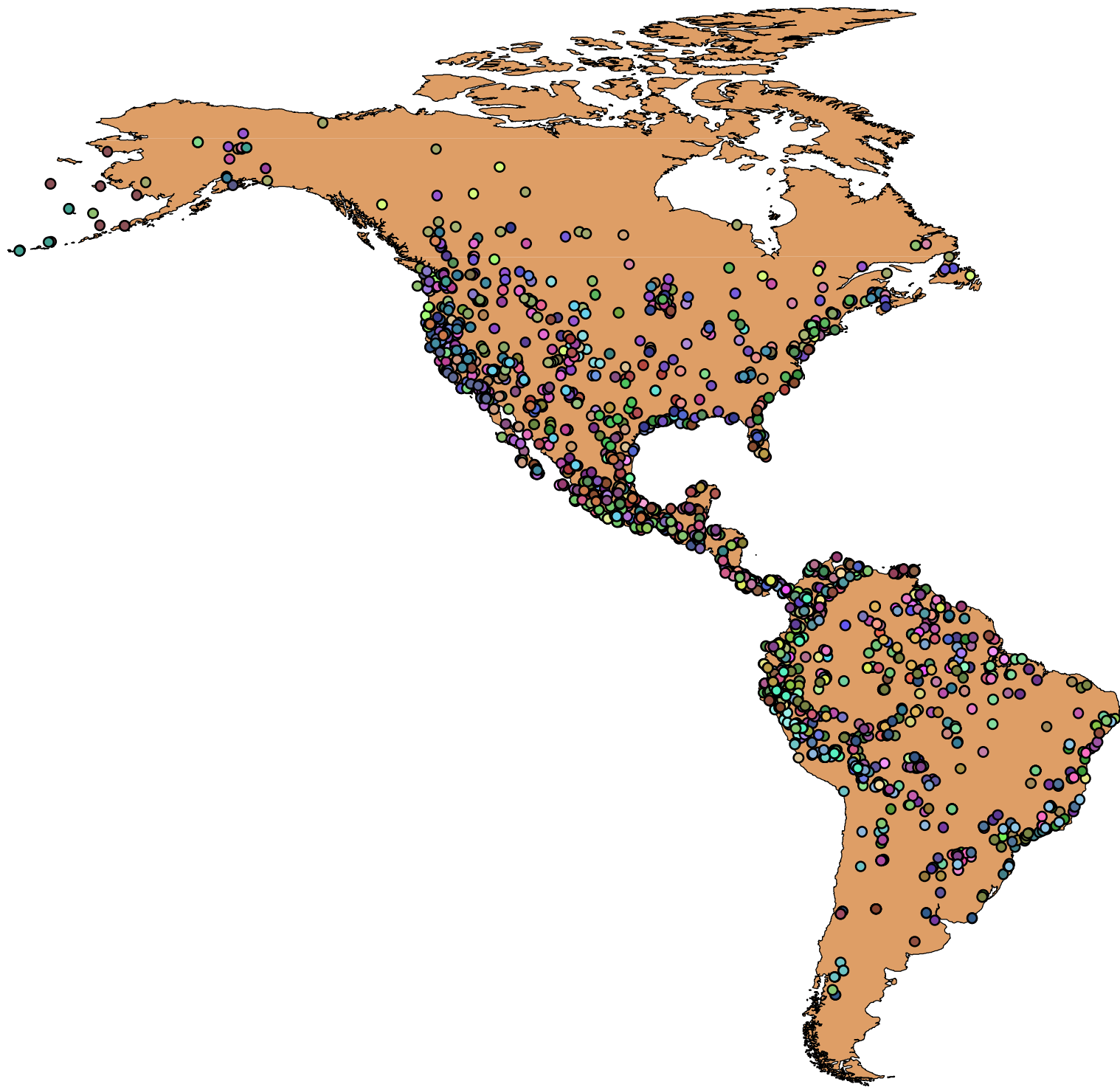

Supplement: S6 Fig — Ten latitude-longitude coordinates were compiled for each species from published records or georeferenced using descriptions of the sampling localities. For the lumped dataset some lineages within the lumped species had less than 10 samples. (PDF) [file pbio.2001073.s006.pdf]

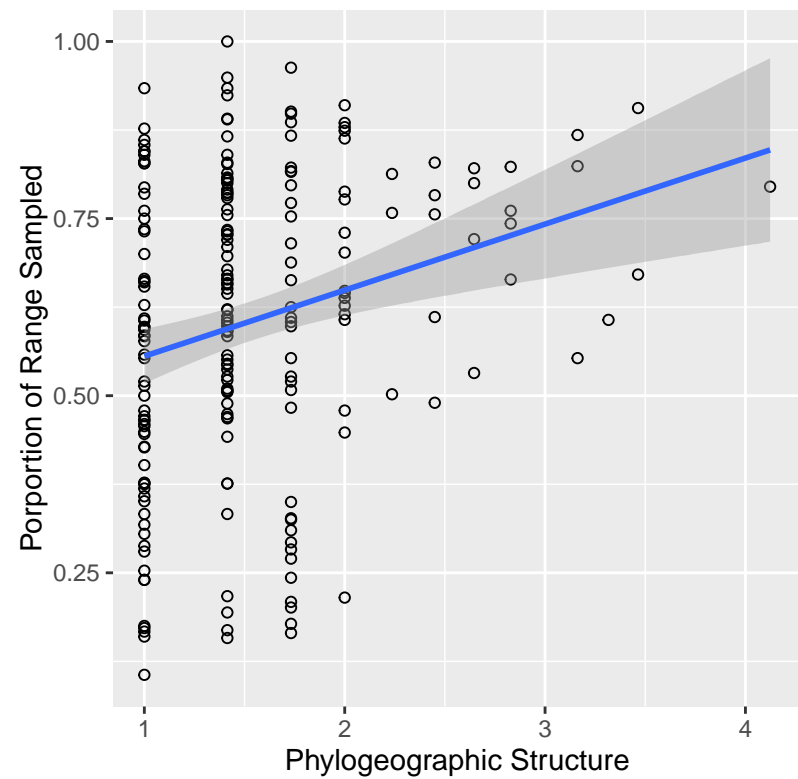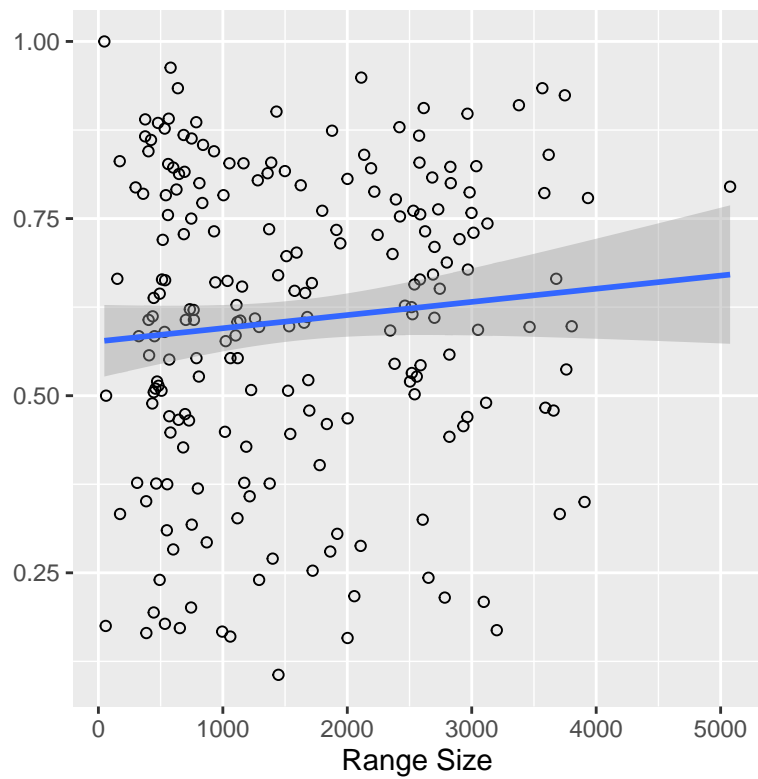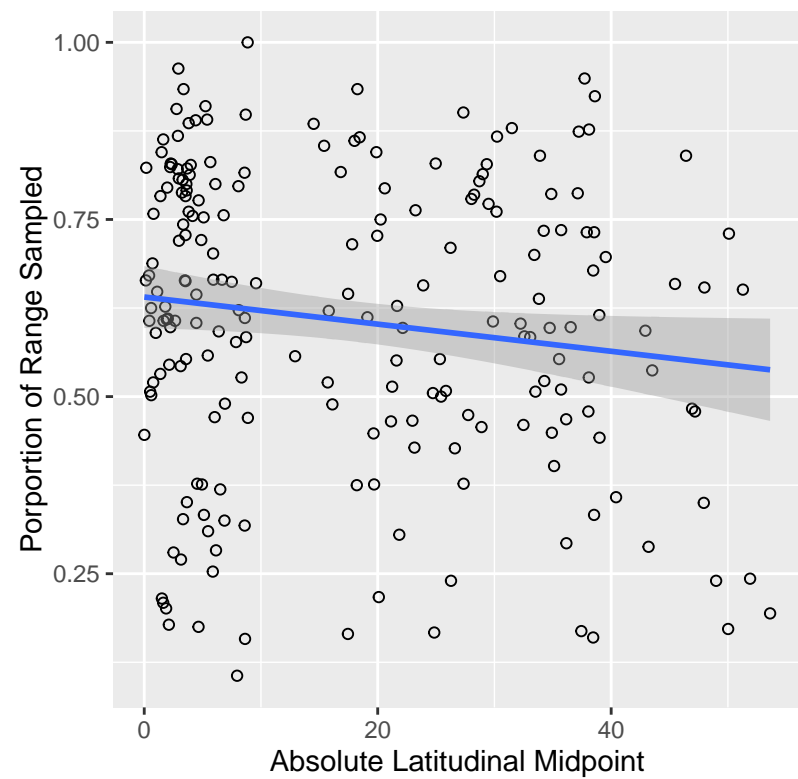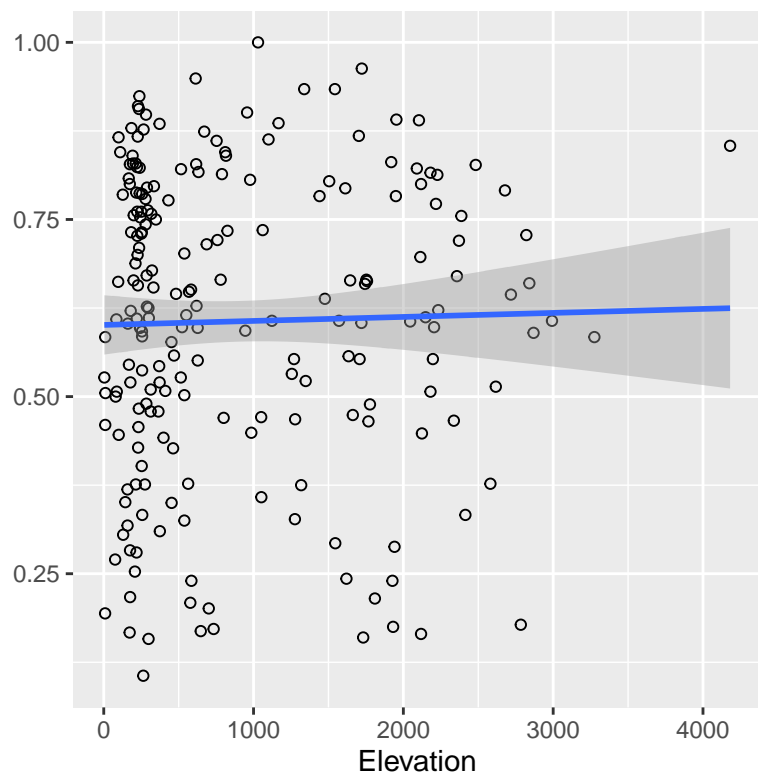

Supplement: S7 Fig — On the y-axis of each plot is the proportion of range size sampled versus phylogeographic structure as determined by the number of bGMYC clusters using a 0.9 threshold (top left), absolute latitudinal midpoint (top right), range size km2 (bottom left), and mean elevational (m) occurrence (bottom right). Summary of regression for each plot is as follows: Phylogeographic Structure: Adjusted R2: 0.059, p-value: 0.0002; Absolute Latitudinal Midpoint: Adjusted R2: 0.015, p-value: 0.043; Range Size: Adjusted R2: 0.004, p-value: 0.174; Elevation: Adjusted R2: -0.004, p-value: 0.74. Blue line and grey shading are the regression line and the 95% CI of the slope, respectively. The underlying data can be found in S2 Table. (PDF) [file pbio.2001073.s007.pdf]
