# Supplementary material for: A latitudinal phylogeographic diversity gradient in birds
Source: PLoS Biol. 2017 Apr 13;15(4):e2001073. doi: 10.1371/journal.pbio.2001073 (PMC5390966; doi:10.1371/journal.pbio.2001073)
Supplement: S1 Table — The expected directionality of the correlation are shown: positive (+) or negative (-). N/A indicate there is no clear indication of how the variables will interact. (DOCX) [file pbio.2001073.s008.docx]

Table S1. Predictions of variable influence on phylogeographic metrics. The expected directionality of the correlation are shown: positive (+) or negative (-). N/A indicate there is no clear indication of how the variables will interact.

| **Description** | **Variable** | **Species Age** | **Phylogeograohic Structure** | **Splitting Rates** | **Lineage Loss** |
| --- | --- | --- | --- | --- | --- |
| Habitat | Temperature | + | + | + | - |
|  | Temperature Seasonality | - | - | +/- | + |
|  | Precipitation | + | + | + | - |
|  | Precipitation Seasonality | +/- | + | + | +/- |
| Habitat Breadth | Temperature Range | + | + | + | - |
|  | Temperature Seasonality Range | + | + | + | - |
|  | Precipitation Range | + | + | + | - |
|  | Precipitation Seasonality Range | +/- | + | + | +/- |
| Habitat Stability | CS Temperature | - | - | +/- | + |
|  | CS Temperature Seasonality | - | - | +/- | + |
|  | CS Precipitation | - | +/- | +/- | + |
|  | CS Precipitation Seasonality | - | +/- | +/- | + |
| Primary Productivity | NPP | + | + | + | - |
| Topography | Elevation | +/- | + | + | +/- |
|  | Elevation Range | +/- | + | + | - |
|  | Ruggedness Index | +/- | + | + | +/- |
| Latitude | Latitude | - | - | - | + |
|  | Latitudinal Range | + | + | + | - |
| Morphology | Hand Wing Index | - | - | - | + |
|  | Tarsus Length | +/- | +/- | +/- | - |
| Behavior | Migratory Distance | - | - | +/- | + |
| History | Species Age | N/A | + | +/- | N/A |
| Geographic Area | Range Size | + | + | - | - |
