# Supplementary material for: A latitudinal phylogeographic diversity gradient in birds
Source: PLoS Biol. 2017 Apr 13;15(4):e2001073. doi: 10.1371/journal.pbio.2001073 (PMC5390966; doi:10.1371/journal.pbio.2001073)
Supplement: S5 Table — Shown are factor loadings, eigenvalues, and the percentage of the variation explained for each of the first four PC axes. (DOCX) [file pbio.2001073.s012.docx]

S5 Table. Principal components analysis output on the 19 climatic variables. Shown are factor loadings, eigenvalues, and percent variation explained for each of the first four axes.

| **Shape Variables** | **PC1** | **PC2** | **PC3** | **PC4** |
| --- | --- | --- | --- | --- |
| Elevation | 0.06 | -0.026 | 0.441 | -0.521 |
| BIO1 = Annual Mean Temperature | -0.282 | -0.194 | -0.152 | -0.062 |
| BIO2 = Mean Diurnal Range (Mean of monthly (max temp - min temp)) | 0.163 | -0.26 | 0.006 | -0.332 |
| BIO3 = Isothermality (BIO2/BIO7) (* 100) | -0.265 | -0.103 | 0.231 | -0.233 |
| BIO4 = Temperature Seasonality (standard deviation *100) | 0.276 | 0.108 | -0.24 | 0.092 |
| BIO5 = Max Temperature of Warmest Month | -0.089 | -0.27 | -0.448 | -0.015 |
| BIO6 = Min Temperature of Coldest Month | -0.301 | -0.143 | 0.015 | -0.005 |
| BIO7 = Temperature Annual Range (BIO5-BIO6) | 0.287 | 0.041 | -0.204 | -0.001 |
| BIO8 = Mean Temperature of Wettest Quarter | -0.177 | -0.101 | -0.31 | -0.327 |
| BIO9 = Mean Temperature of Driest Quarter | -0.237 | -0.218 | 0.054 | 0.141 |
| BIO10 = Mean Temperature of Warmest Quarter | -0.172 | -0.202 | -0.443 | 0.002 |
| BIO11 = Mean Temperature of Coldest Quarter | -0.296 | -0.175 | 0.007 | -0.07 |
| BIO12 = Annual Precipitation | -0.27 | 0.241 | 0.027 | 0.065 |
| BIO13 = Precipitation of Wettest Month | -0.276 | 0.114 | 0.1 | 0.143 |
| BIO14 = Precipitation of Driest Month | -0.132 | 0.404 | -0.173 | -0.208 |
| BIO15 = Precipitation Seasonality (Coefficient of Variation) | -0.081 | -0.377 | 0.23 | 0.226 |
| BIO16 = Precipitation of Wettest Quarter | -0.276 | 0.13 | 0.098 | 0.144 |
| BIO17 = Precipitation of Driest Quarter | -0.146 | 0.403 | -0.159 | -0.187 |
| BIO18 = Precipitation of Warmest Quarter | -0.224 | 0.197 | -0.044 | -0.307 |
| BIO19 = Precipitation of Coldest Quarter | -0.194 | 0.231 | 0.059 | 0.388 |
| Standard deviation | 3.135 | 1.904 | 1.664 | 1.073 |
| Proportion of Variance | 49.2 | 18.1 | 13.8 | 5.8 |
